# Citriquinolinones A and B: Rare Isoquinolinone-Embedded Citrinin Analogues and Related Metabolites from the Deep-Sea-Derived *Aspergillus versicolor* 170217

**DOI:** 10.3390/md21100504

**Published:** 2023-09-23

**Authors:** Shui-Hua Lin, Qing-Xiang Yan, Yong Zhang, Tai-Zong Wu, Zheng-Biao Zou, Qing-Mei Liu, Jia-Yang Jiang, Ming-Min Xie, Lin Xu, You-Jia Hao, Zhu Liu, Guang-Ming Liu, Xian-Wen Yang

**Affiliations:** 1 Department of Pharmacy, Quanzhou Medical College, 2 Anji Road, Quanzhou 362000, China; yxylsh2021@163.com; 2 Key Laboratory of Marine Genetic Resources, Third Institute of Oceanography, Ministry of Natural Resources, 184 Daxue Road, Xiamen 361005, China; youngqx@126.com (Q.-X.Y.); zhangyong@tio.org.cn (Y.Z.); wutaizong@tio.org.cn (T.-Z.W.); zhengbiaozou@njust.edu.cn (Z.-B.Z.); uareal6@163.com (J.-Y.J.); xiemingmin@tio.org.cn (M.-M.X.); xulin@amoytop.com (L.X.); haoyoujia888@163.com (Y.-J.H.); 3 College of Food and Biological Engineering, Jimei University, 43 Yindou Road, Xiamen 361021, China; liuqingmei1229@163.com (Q.-M.L.); gmliu@jmu.edu.cn (G.-M.L.); 4 College of Life Sciences, Hainan University, 58 People’s Avenue, Haikou 570228, China; zhuliu@hainanu.edu.cn

**Keywords:** deep-sea-derived fungus, *Aspergillus versicolor*, theoretical–statistical approaches, citrinin–isoquinolinone hybrid, anti-food allergic activity

## Abstract

A systematic chemical investigation of the deep-sea-derived fungus *Aspergillus versicolor* 170217 resulted in the isolation of six new (**1**–**6**) and 45 known (**7**–**51**) compounds. The structures of the new compounds were established on the basis of exhaustive analysis of their spectroscopic data and theoretical–statistical approaches including GIAO-NMR, TDDFT-ECD/ORD calculations, DP4+ probability analysis, and biogenetic consideration. Citriquinolinones A (**1**) and B (**2**) feature a unique isoquinolinone-embedded citrinin scaffold, representing the first exemplars of a citrinin–isoquinolinone hybrid. Dicitrinones K–L (**3**–**4**) are two new dimeric citrinin analogues with a rare CH-CH_3_ bridge. Biologically, frangula-emodin (**32**) and diorcinol (**17**) displayed remarkable anti-food allergic activity with IC_50_ values of 7.9 ± 3.0 μM and 13.4 ± 1.2 μM, respectively, while diorcinol (**17**) and penicitrinol A (**20**) exhibited weak inhibitory activity against *Vibrio parahemolyticus*, with MIC values ranging from 128 to 256 μM.

## 1. Introduction

Natural products play a dominant role in the discovery of drug leads for the treatment of human diseases [1,2]. Marine organisms living in harsh surroundings, such as high salt, high pressure, and hypoxic environments, are expected to produce unique secondary metabolites when compared with their terrestrial counterparts in terms of structural diversity and functional features, making them a promising storehouse of new bioactive entities for drug leads discovery [3,4]. Of all marine-derived fungi, the genus *Aspergillus* has been the most studied, as it is ubiquitous among almost all ecosystems and it is rich in bioactive secondary metabolites, with multifarious and intricate structures [5]. These include the citrinin family, a compound class that features a 6,8-dihydroxyl-3,4,5-trimethyl-chromene core and exhibits abundant structural diversity by decomposition, dimerization, and trimerization through various pathways [6,7]. Therefore, the biological behaviors of these compounds extend from anticancer to antimicrobial activities, influenza neuraminidase inhibitory and anti-osteoporosis effects. However, citrinin derivatives embedded with alkaloid moiety are rarely reported in the literature, with the exception of citrinidines A–E, whose structures involve a proline-derived unit [8].

**Figure 1:**
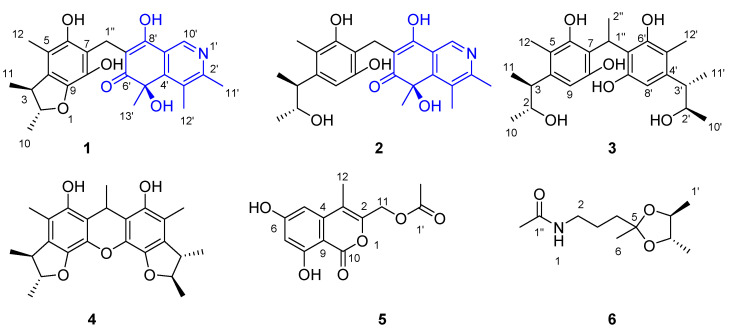
The chemical structures of the new compounds **1**–**6** obtained from *Aspergillus versicolor* 170217.

As part of our ongoing investigation into the chemistry of deep-sea-derived fungi, we obtained an *A. versicolor* fungus from the intestinal contents of a whale *Mesoplodon densirostris* from the East China Sea which was stranded in Ningde, China. The primary chromatographic analysis of the extract from a PDA culture revealed the rich chemical diversity of the metabolites; therefore, we conducted an in-depth investigation of this strain to discover novel and active compounds. As a result, four new dimeric citrinin derivatives (**1**–**4**), one new isochromene derivative (**5**), and one new acetamide (**6**) (Figure 1), together with 45 known compounds, were obtained. The structures of the new compounds were established on the basis of exhaustive analysis of their spectroscopic data and theoretical–statistical approaches including GIAO-NMR, TDDFT-ECD/ORD calculations, DP4+ probability analysis, or biogenetic consideration. Intriguingly, citriquinolinones A–B (**1**–**2**) bear a citrinin scaffold embedded with an unusual isoquinolinone unit, which has not been previously reported in this compound class.

By comparison of the NMR, MS, and optical rotation data with those published in the literature, the known compounds were determined to be viridicatol (**7**) [9], 4-(hydroxymethyl)benzoic acid (**8**) [10], *p*-hydroxybenzoic acid (**9**) [11], ferulic acid (**10**) [12], (2*S*,3*S*)-1-(4-hydroxyphenyl) butane-2,3-diol (**11**) [13], (2*R*,3*S*)-1-(4-hydroxyphenyl)butane-2,3-diol (**12**) [13], 4-((2*S*,3*R*)-3-hydroxybutan-2-yl)-3,6-dimethylbenzene-1,2-diol (**13**) [14,15], phenol A (**14**) [16], monodictyphenone (**15**) [17], dicitrinone F (**16**) [15], diorcinol (**17**) [18], verticilatin (**18**) [19], xerucitrinic acids A (**19**) [20], penicitrinol A (**20**) [21], penicitrinone B (**21**) [22], (+)-austrosene (**22**) [23], 3,6,8-trihydroxy-3,5,7-trimethylisochroman-1-one (**23**) [24], sescandelin (**24**) [25], sescandelin B (**25**) [25], dihydrocitrinone (**26**) [26,27], dihydroxy-3,4,7-trimethylisocoumarin (**27**) [28], lawsozaheer (**28**) [29], dyhydrocitrinin (**29**) [30], citrinin (**30**) [31], 5-methoxysterigmatocystin (**31**) [32], frangula-emodin (**32**) [33], citreorosein (**33**) [34], (*E*)-4-hydroxy-4-(3-hydroxybut-1-en-1-yl)-3,5,5-trimethylcyclohex-2-en-1-one (**34**) [35], citrinal A (**35**) [36], citrinal B (**36**) [36], macrolactin A (**37**) [37], quinolactacin A1 (**38**) [38], 7,8-epoxy-brevianamide Q (**39**) [39], sterigmatocystin (**40**) [40], (+)-brevianamide R (**41**) [39], (−)-brevianamide R (**42**) [39], brevianamide K (**43**) [41], epi-deoxybrevianamide E (**44**) [42], brevianamide W (**45**) [42], 2-(1,1-dimethyl-2-propen-1-yl)-1H-in-dole-3-carboxaldehyde (**46**) [43], 1*H*-indole-3-carbaldehyde (**47**) [44], (1*H*-indol-3-yl) oxoacetamide (**48**) [45], *N*-acetyramine (**49**) [46], cyclo-(Phe-Tyr) (**50**) [47], and glulisine A (**51**) [48]. Herein, we report the isolation, structures, and bioactivities of these isolates.

## 2. Results and Discussion

**Figure 2:**
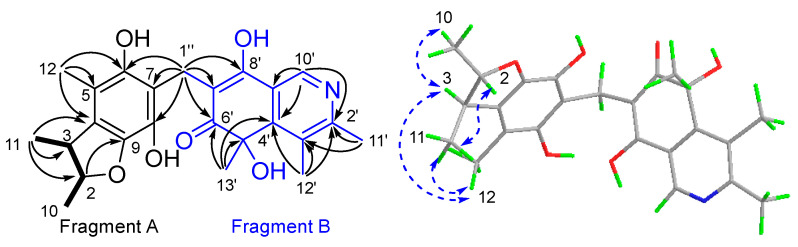
The key ^1^H-^1^H COSY (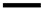), HMBC (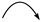), and NOESY (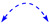) correlations of **1**.

Compound **1** was isolated as a yellow powder. It was assigned a molecular formula C_24_H_27_NO_6_ due to its HRESIMS peak at *m/z* 424.1759 [M−H]^−^, requiring 12 degrees of unsaturation (DOU). The ^1^H and ^13^C NMR spectroscopic data (Figures S1 and S2, Table 1) revealed the presence of six methyls [*δ*_H_ 1.25 (3H, dd, *J* = 6.1 Hz, 1.7 Hz, 10-Me), 1.19 (3H, d, *J* = 6.8 Hz, 11-Me), 2.05 (3H, s, 12-Me), 2.67 (3H, s, 11′-Me), 2.70 (3H, s, 12′-Me), 1.60 (3H, s, 13′-Me); *δ*_C_ 21.2 (q, C-10), 19.8 (q, 11-Me), 12.3 (q, C-12), 20.0 (q, C-11′), 16.7 (q, C-12′), 29.3 (q, C-13′)]; one methylene [*δ*_H_ 3.70 (dd, *J* = 14.2 Hz, 2.4 Hz, H-1″), 3.60 (dd, *J* =14.2 Hz, 4.4 Hz, H_2_-1″); *δ*_C_ 20.2 (t, C-1″)]; three methines [*δ*_H_ 4.30 (dq, *J* = 6.1 Hz, 3.9 Hz, H-2); *δ*_H_ 2.90 (dq, *J* = 6.8 Hz, 3.9 Hz, H-3), 8.86 (s, H-10′); *δ*_C_ 87.6 (d, C-2), 45.3 (d, C-3), 137.6 (d, C-10′)]; fourteen non-hydrogenated carbons, including twelve olefinic [*δ*_C_ 130.4 (s, C-4), 114.0 (s, C-5), 148.0 (s, C-6), 117.6 (s, C-7), 138.2 (s, C-8), 140.9 (s, C-9), 155.8 (s, C-2′), 135.8 (s, C-3′), 159.0 (s, C-4′), 112.6 (s, C-7′), 175.3 (s, C-8′), 128.0 (s, C-9′)], one *sp^3^* [*δ*_C_ 74.7 (s, C-5′)]; and one ketone at *δ*_C_ 196.2 (s, C-6′). Diagnostic HMBC correlations observed from H_3_-11 to C-2/3/4, H_3_-12 to C-4/5/6, H_2_-1″ to C-6/7/8, and from H-2/3 to C-9, together with the COSY cross-peaks H-2/H_3_-10/H-3/H_3_-11, supported a 2,3,5-trimethyl-6,8-dihydrobenzofuran fragment, A [49]. Another fragment, B, was recognized as an isoquinocitrinin owing to the HMBC correlations of H_3_-11′/C-2′/3′; H_3_-12′/C-3′/C-2′ and C-4′, H_3_-13′/C-5′/C-4′ and C-6′, H_3_-10′/C-2′ and C-4′/C-9′; as well as H-1″/C-6′/C-7′/C-8′ [50]. The HMBC cross-peaks to both fragments, starting from H_2_-1″, thus established the CH_2_ linkage between A and B (Figure 2).

**Figure 3:**
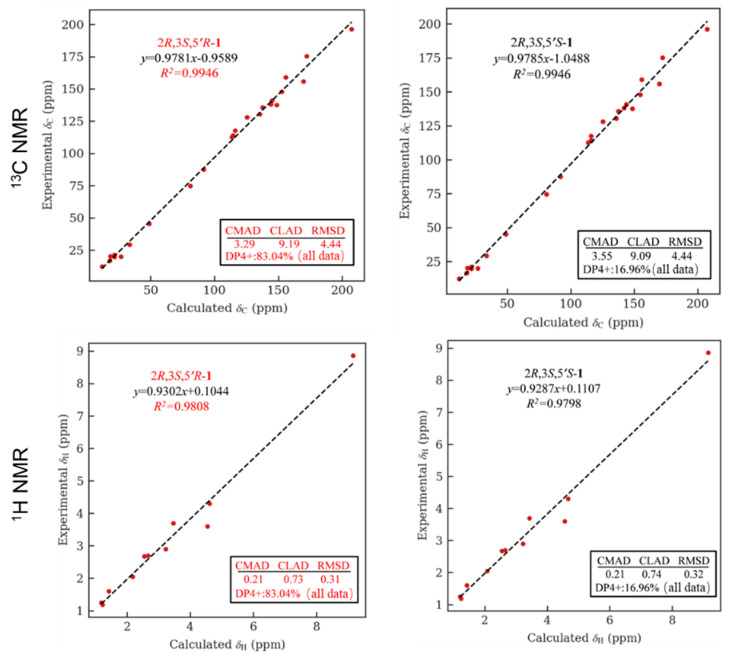
NMR calculation for compound **1**: regression analysis of experimental and calculated ^13^C and ^1^H NMR chemical shifts for 2R*,3S*,5′R*-**1** and 2R*,3S*,5′S*-**1,** with the probability of each isomer analyzed by DP4+ analysis included.

**Figure 4:**
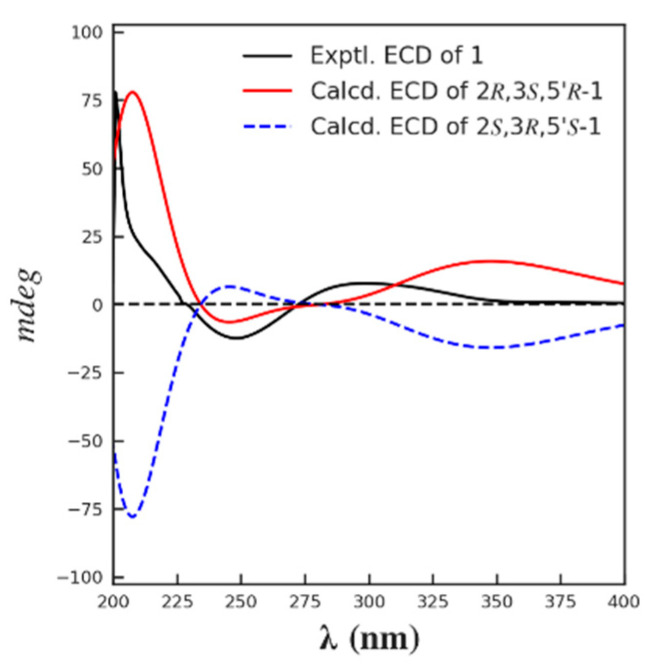
The calculated and the experimental ECD spectra of compound **1**.

The relative configuration of **1** was partially determined by NOESY experiments. The correlations observed from H-2 to H_3_-11 and H-3 to H_3_-10 indicated that 3-Me and 2-Me are *trans*-configured (Figure 2). To furtherly determine the relative configuration between C-5′ and C-2/3, we performed a quantum chemical calculation of NMR data for the two possible isomers of **1**, 2R*,3S*,5′R* and 2R*,3S*,5′S*, along with a DP4+ probability analysis. As a result, the theoretical prediction of the chemical shift of the former showed a better correspondence with the experimental data, with an average probability of 83.04% (Figure 3). Finally, the absolute configuration of **1** was established by a TD-DFT based ECD calculation of the two enantiomers of **1** at the B3LYP/6-31+g (d, p) level, as well as comparison with the experimental data, which indicated the correct configuration as 2*R*,3*S*,5′*R* (Figure 4). Hence, the complete structure of **1** was assigned as shown and named citriquinolinone A. Of note, citriquinolinone A (**1**) represents the first exemplar of an isoquinolinone–citrinin hybrid.

**Figure 5:**
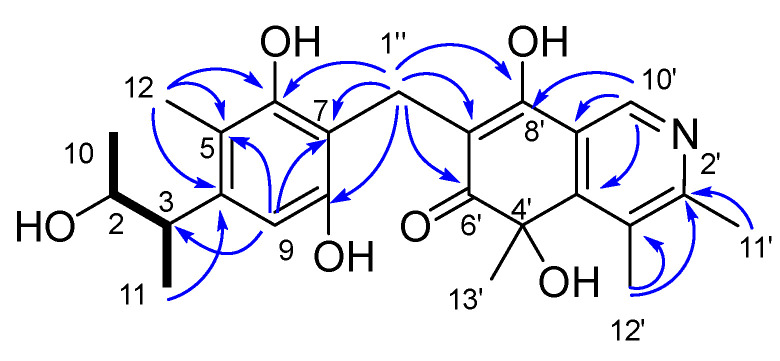
The key ^1^H-^1^H COSY (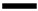) and HMBC (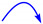) correlations of compound **2**.

Compound **2** was isolated as a yellow amorphous solid and assigned the molecular formula C_24_H_29_NO_6_ on the basis of the [M − H]^−^ ionic peak at *m/z* 426.1996 in its negative HRESIMS spectrum, suggestive of a homologue of **1**. Comparison of NMR data of **2** (Figures S8–S13, Table 1) and **1** revealed a high degree of similarity, including resonances accounting for the isoquinocitrinin substructure, namely fragment B of **1**. The primary differences were attributed to the presence of an aromatic proton (*δ*_H_ 6.30, s; H-9), as well as the shielding of C-2 (Δ*δ*_C_ -5.5). HMBC correlations from H-1″ to C-6/7/8, H-9 to C-5/C-7, together with signals observed from H_3_-11 to C-4 and COSY correlations of H_3_-10/H-2/H-3/-H_3_-11, inferred the penta-substituted benzene fragment A. Considering the molecular formula, the planar structure of **2** was assigned as shown in Figure 5.

**Figure 6:**
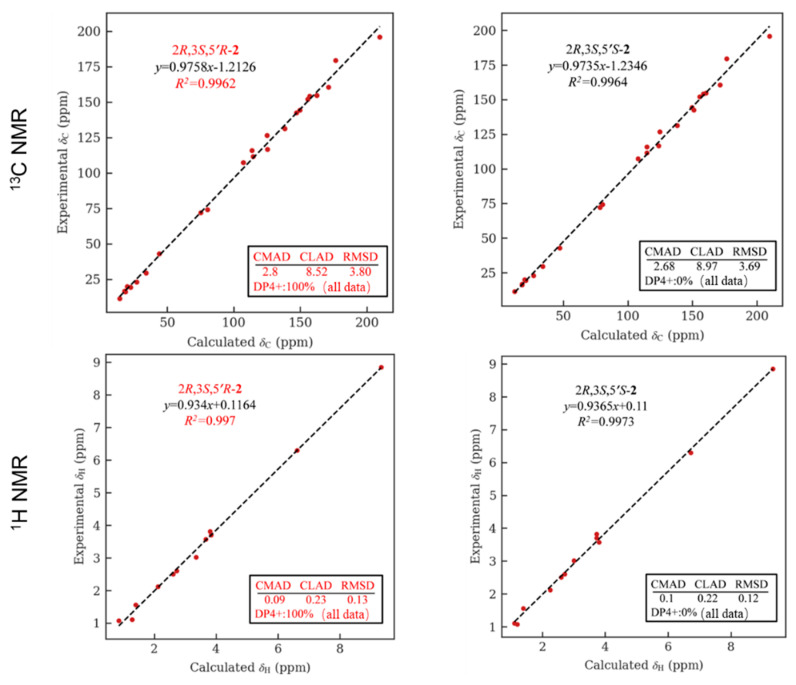
NMR calculation for compound **2**: regression analysis of experimental and calculated ^13^C NMR chemical shifts for 2R*,3S*,5′R*-**2** and 2R*,3S*,5′S*-**2**, with the probability of each isomer analyzed by DP4+ analysis included.

As C-2/3 are not involved in a dihydrofuran ring, in this case, the NOE spectrum is useless for determining its relative configuration. However, compound **2** contains a citrinin moiety similar to that of **1**, namely a phenol A unit; we therefore examined the biogenetic relationship between these two compounds. Capon et al. documented a plausible mechanism for the transformation from citrinin to 2,3,5-trimethyl-6,8-dihydrobenzofuran and phenol A, in which the stereo configurations of C-2/3 were retained in the whole process [27]. This, along with the co-occurrence of citrinin (**30**) and phenol A (**14**) in the same extract and a comparison of the chemical shift of fragment A of **2** with that of **14**, permitted the appearance of relative/absolute configurations of C-2/3 in **2** same as those observed in **1**, **14,** and **30**. To further assign the relative configuration between C-5′and C-2/3, the NMR data of **2** in MeOH was predicted using the GIAO based calculation, followed by DP4+ analysis. The comparison of the theoretical with the experimental data showed that the 2R*,3S*,5′R* isomer exhibited a 100% probability (Figure 6). Likewise, the absolute configuration of **2** was eventually established to be 2*R*,3*S*,5′*R* by comparing the calculated ECD spectrum with that of the experimental data (Figure S42). Therefore, the structure of **2** was assigned, and the compound was given the trivial name citriquinolinone B.

**Figure 7:**
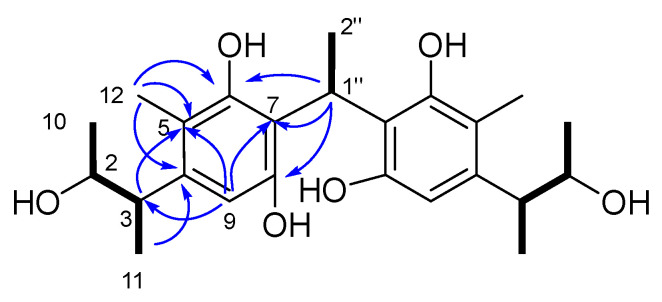
The key ^1^H-^1^H COSY (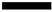) and HMBC (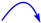) correlations of **3**.

Compound **3** was isolated as a yellow oil and assigned the molecular formula C_24_H_34_O_6_, based on the HRESI(-)MS peak at *m/z* 441.2250 [M+Na]^+^, requiring eight DOU. The ^1^H, ^13^C NMR, and DEPT spectroscopic data (Figures S15–S20 and Table 2) revealed the presence of 24 carbons, including seven methyls, seven methines, and 10 quaternary carbons. In the ^13^C NMR spectrum, all the carbon signals, except the C-1″ (*δ*_C_ 27.7, CH) and C-2″ (*δ*_C_ 17.8, CH_3_) examples, existed in pairs, suggesting that **3** was likely a dimeric compound. The comparison of the 1D and 2D NMR data of **3** with those of **16** showed that they closely resembled each other, with the primary difference being the replacement of a methylene in **2** with a methine and methyl in **3**. Further confirmation was obtained using the COSY correlations of H_3_-10 (*δ*_H_ 1.10, d, *J* = 6.3 Hz)/H-2 (*δ*_H_ 3.80, dq, *J* = 6.5, 6.3 Hz)/H-3 (*δ*_H_ 3.02, dq, *J* = 6.8, 6.5 Hz)/H_3_-11 (*δ*_H_ 1.12, d, *J* = 6.8 Hz), and HMBC cross-peaks from H_3_-12 (*δ*_H_ 2.10, s) to C-4 (*δ*_C_ 143.2 s) C-5 (*δ*_C_ 117.6 s) and C-6 (*δ*_C_ 154.6 s), H_3_-11 to C-4 (*δ*_C_ 143.2 s), H-9 (*δ*_H_ 6.37, s) to C-3 (*δ*_C_ 42.9 s), C-5, and C-7 (*δ*_C_ 116.7 s). The COSY correlations of H-1″ (*δ*_H_ 4.9, m)/H_3_-2″ (*δ*_H_ 1.80, d, *J* = 7.5 Hz), along with the diagnostic HMBC correlations from H-2″ to C-7, H-1″ to C-6, C-7 and C-8 (*δ*_C_ 152.9 s), proved that the linkage between the two monomers were C-1″-2″, thereby assigning the planar structure of **3**, as shown in Figure 7.

**Figure 8:**
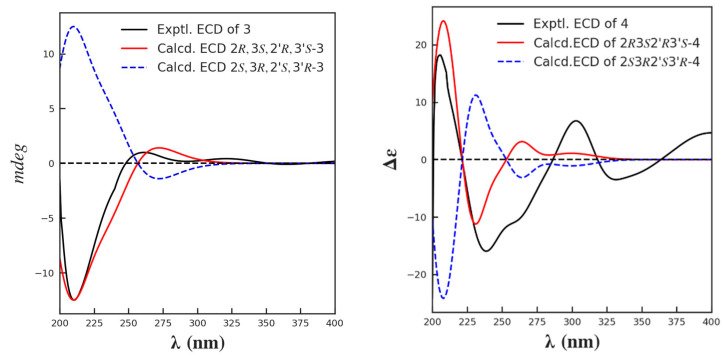
The calculated and the experimental ECD spectra of compounds **3** and **4**.

The relative and absolute configuration of C-2/2′ and C-3/3′ were deduced in the same manner as that used for **1**–**2**, based on a biogenetic consideration with citrinin (**30**) and phenol A (**14**). Finally, by comparison of its calculated and experimental ECD spectra (Figure 8), the absolute configuration of **3** was thus established as 2*R,*3*S,*2′*R,*3′*S*. Herein, **3** was given the trivial name dicitrinone K.

**Figure 9:**
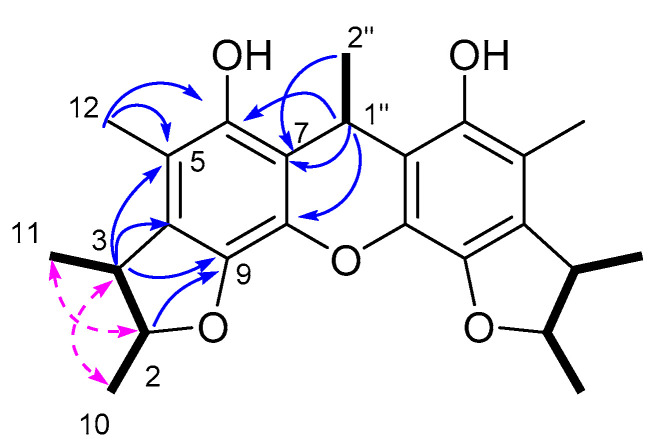
The key ^1^H-^1^H COSY (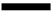), HMBC (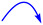) and NOESY (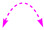) correlations of compound **4**.

Compound **4** was isolated as a yellow amorphous solid. The molecular formula of **4** was deduced as C_24_H_28_O_5_ from the HRESI(−)MS signal *m/z* 395.1841 [M−H]^−^. The ^1^H, ^13^C NMR and DEPT spectroscopic data revealed 24 carbons, including seven methyl singlets, five methines, and 12 protonated carbons. Similar to those of **3**, the carbon signals of **4** also existed in pairs, with the exception of C-1″ (*δ*_C_ 25.6 d) and C-2″ (*δ*_C_ 23.0 q). A detailed comparison of the 1D and 2D NMR data for **4** and **3** revealed that they were closely related, with the major difference being the replacement of an aromatic methine in **3** with a non-hydrogenated carbon (*δ*_C_ 141.9 s; C-9) in **4**. The diagnostic HMBC correlations from H-2 (*δ*_H_ 4.40, m) to C-9 revealed the presence of a dihydrofuran ring. Further connections were confirmed by the COSY correlations of H_3_-10 (*δ*_H_ 1.32, m)/H-2 (*δ*_H_ 4.40, m)/H-3 (*δ*_H_ 3.05, m)/H_3_-11(*δ*_H_ 1.26, m), and H-2″ (*δ*_H_ 1.24, m)/H-1″ (*δ*_H_ 4.48, dd, *J* = 6.7, 13.4 Hz), as well as the HMBC correlations observed from H_3_-12 (*δ*_H_ 2.22, s) to C-4 (*δ*_C_ 130.2 s), C-5 (*δ*_C_ 113.0 s) and C-6 (*δ*_C_ 146.3 s), from H_3_-11 to C-4, from H-3 (*δ*_H_ 3.05, m) to C-4, C-9 (*δ*_C_ 141.9 s), C-5, and from H-1″ to C-7 (*δ*_C_ 116.4 s), C-8 (*δ*_C_ 136.5 s), C-6 (*δ*_C_ 146.3 s) (Figure 9). The relative configuration was determined based on the NOESY correlations of H-2/H_3_-11, H-3/H_3_-10. By comparison of its calculated and experimental ECD spectra, the absolute configuration was established as 2/2′*R*,3/3′*S* (Figure 8), and was given the trivial name dicitrinone L.

**Figure 10:**
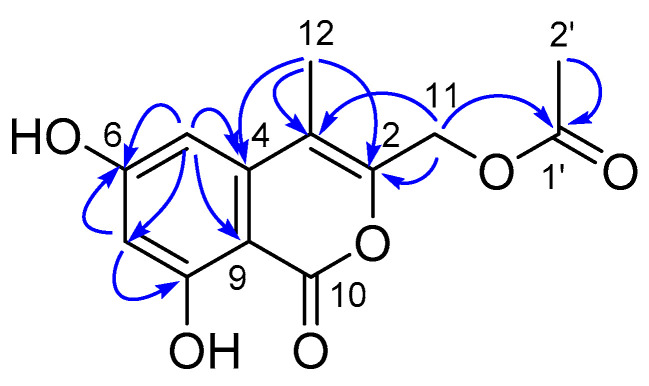
The Key HMBC correlations of compound **5**.

Compound **5** was isolated as a yellow amorphous solid. Its molecular formula was established as C_13_H_12_O_6_, according to the pseudo molecular ion at *m/z* 263.0547 [M−H]^−^ in its HRESI(-)MS spectrum, requiring eight DOU. The ^1^H-NMR and ^13^C NMRDEPT spectroscopic data exhibited 13 carbons (Table 3), including two methyls, one methene, two methines, and eight non-hydrogenated carbons (including one carbonyl and one ketone). According to the key HMBC correlations from H_3_-12 (*δ*_H_ 2.39, m) to C-3 (*δ*_C_ 110.5 s), C-2 (*δ*_C_ 156.4 s) and C-4 (*δ*_C_ 140.7 s), H_3_-2′ (*δ*_H_ 2.04, s) to C-1′ (*δ*_C_ 172.5 s), H_2_-11 (*δ*_H_ 5.13, s) to C-2, C-3 and C-1′, H-7 (*δ*_H_ 6.34, d, *J* = 1.6 Hz) to C-6 (*δ*_C_ 167.5 s), C-8 (*δ*_C_ 165.3 s), C-5 (*δ*_C_ 102.6 s) and C-9 (*δ*_C_ 99.7 s), H-5 (*δ*_H_ 6.49, d, *J* = 1.6 Hz) to C-6, C-3, C-7 (*δ*_C_ 101.8 d) and C-9, along with the consideration of the rest of the DOU, the structure of **5** was assigned, as shown in Figure 10. Therefore, **5** was identified as (6,8-dihydroxy-4-methyl-1-oxo-1*H*-isochromen-3-yl) methyl acetate.

**Figure 11:**
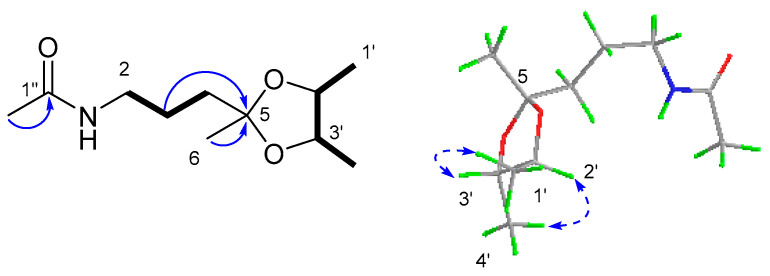
The key ^1^H-^1^H COSY (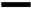), HMBC (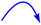) and NOESY (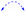) correlations of compound **6**.

Compound **6** was obtained as a yellow oil. Its molecular formula was established as C_11_H_21_NO_3_ on the basis of the pseudo molecular ion at *m/z* 238.1435 [M + Na]^+^ in its HRESI(+)MS spectrum, requiring two degrees of unsaturation. The ^13^C NMR spectrum, in association with the DEPT spectrum, indicated 11 carbon signals ascribed to four methyls (*δ*_C_ 23.3 q, C-2″; 17.2 q, C-1′; 16.4 q, C-4′; and 25.8 q, C-6), two *sp^3^* methylenes (*δ*_C_ 37.6 t, C-4; 23.7 t, C-3; and 39.6 t, C-2), two *sp^3^* methines (*δ*_C_ 78.0 d, C-2′ and *δ*_C_ 79.0 d, C-3′), one *sp^3^* quaternary carbon (*δ*_C_ 108.8 s, C-5), and one carbonyl (*δ*_C_ 170.0 s, C-1″). The 2D NMR spectra revealed COSY correlations of H_2_-2 (*δ*_H_ 3.25, dd, *J* = 12.7, 6.4 Hz)/H_2_-3 (1.67, m; 1.62, m)/H_2_-4 (1.66, m), H_3_-1′ (*δ*_H_ 1.23, d, *J* = 5.8 Hz)/H-2′ (*δ*_H_ 3.68, m)/H-3′ (*δ*_H_ 3.56, m)/H-4′ (*δ*_H_ 1.24, d, *J* = 5.7 Hz). These, along with the HMBC correlation from H_2_-3 and H_3_-6 (*δ*_H_ 1.30, s) to C-5, allowed for the assignment of the planar structure, as shown in Figure 11. For the stereochemistry, the key NOESY correlations of H_3_-1′/H-3′ and H_3_-4′/H-2′ established a *trans*-relationship between the two methyls. Additionally, a DFT calculation of the OR value of the two possible enantiomers was performed, in which the predicted OR of the 2’*S*3’*S* isomer showed the same sign as the experimental result (Table 4). Therefore, **6** was determined as *N*-(3-((2′S,3′S)-2′,3′,5′-trimethyl-1,3-dioxolan-2-yl)propyl)acetamide.

All isolates were tested for anti-food allergic activity under the concentration of 50 μM and for antibacterial activity up to 256 µM. As a result, diorcinol (**17**) and frangula-emodin (**32**) showed potent degranulation-inhibitory activity, with IC_50_ values of 13.4 and 7.9 μM, respectively, while diorcinol (**17**) and penicitrinol A (**20**) exhibited mild inhibition against *Vibrio parahemolyticus*, with MIC values of 128 μM and 256 μM, respectively (Table 5). No compounds were active against MRSA (*methicillin-resistant Staphylococcus aureus*).

## 3. Materials and Methods

### 3.1. General Experimental Procedures

NMR spectra were recorded on a Bruker 400 MHz spectrometer. The HRESIMS spectra were recorded on a Waters Xevo G2 Q-TOF mass spectrometer (Waters Corporation, Milford, MA, USA). Optical rotations were obtained with an Anton Par polarimeter (MCP100). ECD spectra were measured on a Chirascan spectrometer. The semi-preparative HPLC was conducted on an Agilent instrument (1260) equipped with a 1260 Diode Array Detector (DAD) and column COSMOSIL 5 C_18_-MS-II 10 ID × 250 mm (Nacalai Tesque, Japan). Column chromatography was performed on silica gel, Sephadex LH-20, and ODS. Analytical-grade solvents, purchased from Sinopharm Chemical Reagent Co., Ltd. or Xilong Scientific Co., Ltd., were used for solvent extractions Chromatography solvents were HPLC grade, supplied by Xilong Scientific Co., Ltd. or Sigma-Aldrich. Deuterated solvents were purchased from Cambridge Isotopes.

### 3.2. Fungal Identification, Fermentation, and Extraction

The fungus strain 170217 was isolated from the intestinal contents of a whale *Mesoplodon densirostris* stranded in Ningde of the East China Sea. It was identified to be an *Aspergillus versicolor* (GenBank accession number SUB13826338), as the 18S rRNA gene sequence alignment demonstrated that it was 100% identical to the *Aspergillus versicolor* TF34 (GenBank accession number MN515366.1). For scale-up fermentation, the *A. versicolor* 170217 was grown under static conditions at 25 °C in 85 × 1 L Erlenmeyer flasks, each containing 200 g oatmeal agar, including 100 g of oatmeal, 15% sea salt, and 120 mL of distilled H_2_O. After 30 days, the fermentation product was extracted, in triplicate, using 95% ethanol. Then, the organic solvent was combined and concentrated to a small volume. The latter was then extracted, in triplicate, using ethyl acetate. Finally, the solvent was removed under vacuum to provide the crude extract (153 g).

### 3.3. Isolation and Purification

The crude extract was separated into four fractions (Fr.1−Fr.4) *via* medium pressure liquid chromatography (MPLC, 680 mm × 58 mm) on silica gel, with a gradient of CH_2_Cl_2_-MeOH (100%→85%). Fraction Fr.2 (17 g) was separated into eight subfractions (Fr.2-1–Fr.2-8) by column chromatography (CC) over ODS (460 mm × 26 mm; MeOH-H_2_O, 30%→100%). Subfraction Fr.2-1 was purified by CC over Sephadex LH-20 (MeOH) and HPLC (MeOH-H_2_O, 45%→100%) to yield **5** (1.5 mg, 0.001%), **36** (19 mg, 0.012%), and **43** (2 mg, 0.001%). Subfraction Fr.2-2 was separated by CC over Sephadex LH-20 (MeOH) and CC on silica gel, followed by HPLC (MeOH-H_2_O, 55%→100%) to afford **27** (2.4 mg, 0.002%). Compound **35** (12 mg, 0.008%) was isolated from Fr.2-3 by CC over Sephadex LH-20 (MeOH) and HPLC (MeOH-H_2_O, 55%→100%). Subfraction Fr.2-4 was separated by CC over Sephadex LH-20 (MeOH) to obtain **30** (90 mg, 0.059%) and **20** (1 mg, 0.0006%). Subfraction Fr.2-5 was separated by CC over Sephadex LH-20 (MeOH) to yield **31** (6 mg, 0.0039%), and **32** (3mg, 0.002%) was isolated from Fr.2-6 by CC over Sephadex LH-20 (MeOH). Compound **4** (3 mg, 0.002%) was isolated from Fr.2-7 by CC over Sephadex LH-20 (MeOH) and HPLC (MeOH-H_2_O, 60%→100%). Subfraction Fr.2-8 was separated by CC over Sephadex LH-20 (MeOH) and HPLC (MeOH-H_2_O, 40%→100%) to obtain **17** (3 mg, 0.002%), **44** (15 mg, 0.01%), **45** (9 mg, 0.006%), and **46** (2.3 mg, 0.002%).

Fraction Fr.3 (42 g) was separated into 21 subfractions by CC over ODS (460 mm × 26 mm; MeOH-H_2_O, 10%→100%). Subfraction Fr.3-3 was purified by CC over Sephadex LH-20 (MeOH) and silica gel to yield **6** (5.8 mg, 0.045%), **13** (11 mg, 0.007%), **26** (22 mg, 0.014%), **10** (4 mg, 0.003%), and **48** (10 mg, 0.006%). Subfraction Fr.3-7 was purified by CC over Sephadex LH-20 (MeOH) and HPLC (MeOH-H_2_O, 60%→100%) to yield compound **1** (3 mg, 0.002%). Subfraction Fr.3-5 was purified by CC over Sephadex LH-20 (MeOH) and silica gel to yield **23** (4 mg, 0.003%) and **39** (1.5 mg, 0.001%). Compound **21** (4 mg, 0.003%) was obtained from Fr.3-6 by CC over Sephadex LH-20 (MeOH), **18** (2 mg, 0.001%) and **19** (2 mg, 0.001%) were obtained from Fr.3-11 by CC over Sephadex LH-20 (MeOH), and HPLC. Subfraction Fr.3-9 was purified by CC over Sephadex LH-20 (MeOH) and silica gel to yield **33** (4 mg, 0.003%), **37** (36 mg, 0.023%), **40** (12 mg, 0.008%), **41** (32 mg, 0.021%), and **42** (4 mg, 0.003%). Subfraction Fr.3-4 was purified by CC over Sephadex LH-20 (MeOH), silica gel, and HPLC to yield **24** (1.5 mg, 0.001%), **29** (76 mg, 0.05%), and **47** (4 mg, 0.003%).

Fraction Fr.4 (11.8 g) was separated into seven subfractions (Fr.4-1-Fr.4-7) by CC over ODS (460 mm × 26 mm; MeOH-H_2_O, 10%→100%). Subfraction Fr.4-1 was purified by CC over Sephadex LH-20 (MeOH) and silica gel to yield **8** (2.3 mg, 0.002%), **9** (18 mg, 0.012%), **11** (8 mg, 0.005%), **12** (2 mg, 0.001%), and **49** (3.5 mg, 0.002%). Subfraction Fr.4-2 was purified by CC over Sephadex LH-20 (MeOH) and HPLC to yield **28** (3.3 mg, 0.002%) and **7** (2 mg, 0.001%). Compound **15** (30 mg, 0.02%) was isolated from Fr.4-3 by CC over Sephadex LH-20 (MeOH). Compounds **14** (5 mg, 0.003%), **50** (2 mg, 0.001%), and **51** (26 mg, 0.017%) were isolated from Fr.4-4 by CC over Sephadex LH-20 (MeOH) and silica gel. Compound **25** (2.7 mg, 0.002%) was isolated from Fr.4-5 by CC over Sephadex LH-20 (MeOH). Subfraction Fr.4-6 was repeatedly separated by CC over Sephadex LH-20 (MeOH), silica gel to provide **2** (5 mg, 0.003%). Subfraction Fr.4-7 was subjected toCC over Sephadex LH-20 (MeOH). Final purification by HPLC afforded **16** (52.8 mg, 0.038%) and **3** (10 mg, 0.006%).

Citriquinolione A (**1**): Yellow powder; [*α*${]}_{D}^{20}$ +44 (c 0.1, MeOH); UV (MeOH) *λ*max (logε) 271 (2.86) nm, 202 (3.43) nm; ECD (MeOH) (Δε) 252 (−3.18), 297 (+1.92) nm; ^1^H and ^13^C NMR data; see Table 1; HRESIMS *m/z* 424.1759 [M−H]^−^ (calcd for C_24_H_26_NO_6_, 424.1760, ΔmDa −0.1).

Citriquinolione B (**2**): Yellow amorphous solid; [*α*${]}_{D}^{20}$ −18 (*c* 0.15, MeOH); UV (MeOH) *λ*max (logε) 310 (2.10) nm, 351 (2.40) nm; ECD (MeOH) (Δε) 212 (−5.51), 285 (+ 0.40) nm; ^1^H and ^13^C NMR data; see Table 1; HRESIMS *m/z* 426.1996 [M−H]^−^ (calcd for C_24_H_28_NO_6_, 426.1917, ΔmDa 7.9).

Dicitrinone K (**3**): Yellow oil; [*α*${]}_{D}^{20}$ −26 (*c* 0.1, MeOH); UV (MeOH) *λ*max (logε) 278 (2.05) nm; ECD (MeOH) (Δε) 210 (−3.17), 261 (+ 0.25) nm; ^1^H and ^13^C NMR data; see Table 2; HRESIMS *m/z* 441.2250 [M+Na]^+^ (calcd for C_24_H_33_O_6_, 441.2253, ΔmDa −0.3).

Dicitrinone L (**4**): Yellow amorphous solid; [*α*${]}_{D}^{20}$ +42 (*c* 0.1, MeOH); UV(MeOH) *λ*max (logε) 302 (2.56) nm; ECD (MeOH) (Δε) 239 (−3.82), 303 (+ 1.62) nm; ^1^H and ^13^C NMR data; see Table 2; HRESIMS *m/z* 395.1841 [M−H]^−^ (calcd for C_24_H_28_O_5_, 395.1858, ΔmDa −1.7).

(6,8-Dihydroxy-4-methyl-1-oxo-1*H*-isochromen-3-yl) methyl acetate (**5**): Yellow amorphous solid; ^1^H and ^13^C NMR data, see Table 3; HRESIMS *m/z* 263.0547 [M−H]^−^ (calcd for C_13_H_11_O_6_, 263.0556, ΔmDa −0.9).

*N*-(3-((2′S,3′S)-2′,3′,5′-Trimethyl-1,3-dioxolan-2-yl)propyl)acetamide (**6**): yellow oil; [*α*${]}_{D}^{20}$ +18 (*c* 0.1, MeOH); ^1^H and ^13^C NMR data; see Table 4; HRESIMS *m/z* 238.1435 [M+Na]^+^ (calcd for C_11_H_21_NO_3_, 238.1419, ΔmDa 1.6).

### 3.4. Theoretical Calculations

Conformational analysis was first performed via random searching in the stochastic algorithm using the MMFF94 force field, with an energy cutoff of 7.0 kcal/mol and an RMSD threshold of 0.5 Å. The predominant conformers were relocated and confirmed at the B3LYP/6-31G(d) level. The theoretical ECD spectra were calculated using the time-dependent density functional theory (TD-DFT) in methanol. The ECD spectrum was obtained by averaging each conformer using the Boltzmann distribution theory. NMR calculation and conformational optimization were performed under the HF/6-31G(d) level; then NMR was calculated at the mPW1PW91/6-311G (2d, p) level. For ORD calculation, the conformations of the compounds were optimized at the B3LYP/6-31G(d) level to obtain the energy-minimized conformers. Then, the optimized conformers were subjected to the calculations of specific rotation value using the B3LYPspAug-cc-pVDZ level (λ = 589.3 nm). The calculated specific rotations were later obtained according to the Boltzmann weighting of each conformer.

### 3.5. Anti-Food Allergic Bioassay

The in vitro anti-food allergic assay was conducted following our previous protocol [51,52]. Briefly, the rat basophilic leukemia 2H3 (RBL-2H3) cells were incubated with dinitrophenyl (DNP)-immunoglobulin E (IgE) overnight. Then, the IgE-sensitized RBL-2H3 cells were pretreated with the tested compounds and stimulated with DNP-bovine serum albumin (BSA). The bioactivity was quantified by measuring the fluorescence intensity of the hydrolyzed substrate in a fluorometer. Loratadine, a commercially available anti-allergy medicine, was used as a positive control.

### 3.6. Antibacterial Bioassay

The antibacterial assay (MICs) was evaluated by a broth microdilution in 96-well plates, and two bacterial strains, MRSA and *Vibrio parahemolyticus*, were used as the test targets, following the methods included in the literature [53]. The tested compounds were prepared in 20% DMSO to obtain the mother solution with the initial concentration of 20 mM, and then were diluted 40-fold with PBS. The compound solution was subsequently diluted using the 2× dilution method in series to reach eight concentrations from 512 µM to 4 µM. The bacteria are diluted into 5 × 10^5^ CFU/mL and added into the 96-well plates, and equal volumes of the compound solutions were added into each well. After culturing for 24 h at 37 °C, the plates were observed by the naked eye. Each experiment was repeated three times. An equal amount of DMSO was used as a negative control.

## 4. Conclusions

In the present study, the chemical investigation of the deep-sea-derived fungus *A. versicolor* 170217 led to the isolation of six new (**1**–**6**) and 45 known (**7**–**51**) compounds, enriching the diversity of secondary metabolites from the deep-sea-derived *Aspergillus*. The structures, including absolute configurations of new compounds, were elucidated by the analysis of comprehensive spectroscopic data, quantum chemical calculations, and biogenetic considerations. Biologically, compounds **32** and **17** showed remarkable anti-food allergic activity, with IC_50_ values of 7.9 ± 3.0 μM and 13.4 ± 1.2 μM, respectively, while displaying no cytotoxicity. Moreover, diorcinol (**17**) and penicitrinol A (**20**) exhibited mild inhibition against *Vibrio parahemolyticus*, with MIC values of 128 μM and 256 μM, respectively.

## Figures and Tables

**Table 1:** ^1^H (400 Hz) and ^13^C (100 Hz) NMR spectroscopic data of compounds **1** and **2** (*δ* in ppm, *J* in Hz within parentheses).

No.	1		2	
	*δ*_C_	*δ*_H_	*δ*_C_	*δ*_H_
2	87.6 CH	4.30 (dq, 6.1, 3.9)	72.1 CH	3.82 (dq, 6.5, 6.4)
3	45.3 CH	2.90 (dq, 6.8, 3.9)	43.0 CH	3.02 (dq, 6.9, 6.5)
4	130.4 C		142.5 C	
5	114.0 C		116.7 C	
6	148.0 C		154.7 C	
7	117.6 C		115.8 C	
8	138.2 C		154.3 C	
9	140.9 C		107.3 CH	6.30 s
10	21.2 CH_3_	1.25 (dd, 1.7, 6.1)	19.4 CH_3_	1.08 (d, 6.4)
11	19.8 CH_3_	1.19 (d, 6.8)	16.2 CH_3_	1.11 (d, 6.9)
12	12.3 CH_3_	2.05 s	11.5 CH_3_	2.12 s
1″	20.2 CH_2_	3.70 (dd, 14.2, 2.4) 3.60 (dd, 14.2, 4.4)	20.0 CH_2_	3.57 (d, 14.2) 3.70 (d, 14.2)
10′	137.6 CH	8.86 s	144.4 CH	8.85 s
11′	20.0 CH_3_	2.67 s	23.0 CH_3_	2.51 s
12′	16.7 CH_3_	2.70 s	16.8 CH_3_	2.61 s
13′	29.3 CH_3_	1.60 s	29.6 CH_3_	1.56 s
2′	155.8 C		160.6 C	
3′	135.8 C		131.3 C	
4′	159.0 C		152.1 C	
5′	74.7 C		74.2 C	
6′	196.2 C		195.9 C	
7′	112.6 C		111.7 C	
8′	175.3 C		179.6 C	
9′	128.0 C		126.7 C	

Recorded in CD_3_OD.

**Table 2:** ^1^H (400 Hz) and ^13^C (100 Hz) NMR data for compounds **3** and **4** (δ in ppm, *J* in Hz within parentheses).

No.	3		4	
	δ_C_	δ_H_	δ_C_	δ_H_
12/12’	11.6 CH_3_	2.10 s	11.7 CH_3_	2.22 s
11/11’	16.5 CH_3_	1.12 (d, 6.8)	20.0 CH_3_	1.26 m
10/10’	19.6 CH_3_	1.10 (d, 6.3)	21.3 CH_3_	1.32 m
1’’	27.7 CH	4.90 m	25.6 CH	4.48 (dd, 13.4, 6.7)
2’’	17.8 CH3	1.80 (d, 7.5)	23.0 CH_3_	1.24 m
3/3’	42.9 CH	3.02 (dq, 6.8, 6.5)	45.4 CH	3.05 m
2/2’	72.1 CH	3.80 (dq, 6.5, 6.3)	88.1 CH	4.40 m
4/4’	143.2 C		130.2 C	
5/5’	117.6 C		113.0 C	
6/6’	154.6 C		146.3 C	
7/7’	116.7 C		116.4 C	
8/8’	152.9 C		136.5 C	
9/9’	106.7 CH	6.37 s	141.9 C	

Recorded in CD_3_OD.

**Table 3:** ^1^H (400 Hz) and ^13^C (100 Hz) NMR data for compounds **5** and **6** (*δ* in ppm, *J* in Hz within parentheses).

No.	5 ^a^		6 ^b^	
	δ_C_	δ_H_	δ_C_	δ_H_
2	156.4 C		39.6 CH_2_	3.25 (dd, 12.7, 6.4)
3	110.5 C		23.7 CH_2_	1.67 m, 1.62 m
4	140.7 C		37.6 CH_2_	1.66 m
5	102.6 CH	6.49 (d, 1.6)	108.8 C	
6	167.5 C		25.8 CH_3_	1.30 s
7	101.8 CH	6.34 (d, 1.6)		
8	165.3 C			
9	99.7 C			
10	166.8 C			
11	60.2 CH_2_	5.13 s		
12	17.2 CH_3_	2.39 s		
1′	172.5 C		17.2 CH_3_	1.23 (d, 5.8)
2′	20.7 CH_3_	2.04 s	78.0 CH	3.68 m
3′			79.0 CH	3.56 m
4′			16.4 CH_3_	1.24 (d, 5.7)
1″			170.0 C	
2″			23.3 CH_3_	1.95 s

^a^ Recorded in CD_3_OD; ^b^ Recorded in CDCl_3._

**Table 4:** The calculated and experimental specific rotation values of compound **6**.

Compounds/Isomers	Specific Rotation (°)	Energy (Hartree)	Energy (kcal/mol)	Population (%)
**6**	+18	-	-	-
2′*R*, 3′*R*-**6A**	−236	−712.34661	0.29	36
2′*R*, 3′*R*-**6B**	−21	−712.3448059	1.42	5.3
2′*R*, 3′*R*-**6C**	−112	−712.3470707	0	58.7

**Table 5:** Bioactivities of compounds from *Aspergillus versicolor* 170217.

Compounds	IC_50_ (µM) ^a^	Compounds	MIC (µM) ^b^
**17**	13.4 ± 1.2	**17**	128
**32**	7.9 ± 3.0	**20**	256
Others	-	Others	-
Loratadine *	89.9	Vancomycin *	>32

^a^ anti-food allergic activity; ^b^ anti-*Vibrio parahemolyticus* activity; - inactive; * positive control.

## Supplementary Materials

The following are available online at https://www.mdpi.com/article/10.3390/md21100504/s1, One-dimensional and two-dimensional NMR spectra of all new compounds, as well as the experimental and calculated ECD spectra of compound **2**.
