# Supplementary material for: Citriquinolinones A and B: Rare Isoquinolinone-Embedded Citrinin Analogues and Related Metabolites from the Deep-Sea-Derived Aspergillus versicolor 170217
Source: Mar Drugs. 2023 Sep 23;21(10):504. doi: 10.3390/md21100504 (PMC10608187; doi:10.3390/md21100504)

Supporting Information

Citriquinolinones A and B: Rare Isoquinolinone-Embedded Citrinin Analogues and Related Metabolites from the Deep-Sea-Derived *Aspergillus versicolor* 170217

Shui-Hua Lin [1,2,†], Qing-Xiang Yan [2,†], Yong Zhang [2], Taizong, Wu,[2] Zheng-Biao Zou [2], Qing-Mei Liu [3], Jia-Yang Jiang [2,4], Ming-Min Xie [2], Lin Xu [2], You-Jia Hao [2], Zhu Liu [4], Guang-Min Liu [3] and Xian-Wen Yang [2,*]

[1] Quanzhou Medical College, 2 Anji Road, Quanzhou 362000, China
[2] Key Laboratory of Marine Genetic Resources, Third Institute of Oceanography, Ministry of Natural Resources, 184 Daxue Road, Xiamen 361005, China
[3] College of Food and Biological Engineering, Jimei University, 43 Yindou Road, Xiamen 361021, Fujian, China
[4] College of Life Sciences, Hainan University, 58 People's Avenue, Haikou, Hainan 570228, China

Figure S1. ^{1}H NMR spectrum (400 MHz, CD_3OD) of compound **1**.

Figure S2. ^{13}C NMR and DEPT spectra (100 MHz, CD_3OD) of compound **1**.

Figure S3. HMQC spectrum (400 MHz, CD_3OD) of compound **1**.

Figure S4. ^{1}H-^{1}H COSY spectrum (400 MHz, CD_3OD) of compound **1**.

Figure S5. HMBC spectrum (400 MHz, CD_3OD) of compound **1**.

Figure S6. NOESY spectrum of (400 MHz, CD3OD) compound **1**.

Figure S7. HR-ESI-MS of compound **1**.

Figure S8. 1H NMR spectrum (400 MHz, CD3OD) of compound **2**.

Figure S9. 13C NMR and DEPT spectra (100 MHz, CD_3OD) of compound **2**.

Figure S10. HMQC spectrum (400 MHz, CD_3OD) of compound **2**.

Figure S11. ^{1}H-^{1}H COSY spectrum (400 MHz, CD_3OD) of compound **2**.

Figure S12. HMBC spectrum (400 MHz, CD_3OD) of compound **2**.

Figure S13. NOESY spectrum of (400 MHz, CD_3OD) compound **2**.

Figure S14. HR-ESI-MS of compound **2**.

Figure S15. 1H NMR spectrum (400 MHz, CD3OD) of compound **3**.

Figure S16. 13C NMR and DEPT spectra (100 MHz, CD_3OD) of compound **3**.

Figure S17. HMQC spectrum (400 MHz, CD_3OD) of compound **3**.

Figure S18. ^{1}H-^{1}H COSY spectrum (400 MHz, CD_3OD) of compound **3**.

Figure S19. HMBC spectrum (400 MHz, CD_3OD) of compound **3**.

Figure S20. NOESY spectrum of (400 MHz, CD_3OD) compound **3**.

Figure S21. HR-ESI-MS of compound **3**.

Figure S22. 1H NMR spectrum (400 MHz, CD_3OD) of compound **4**.

Figure S23. ^{13}C NMR and DEPT spectra (100 MHz, CD_3OD) of compound **4**.

Figure S24. HMQC spectrum (400 MHz, CD_3OD) of compound **4.**

Figure S25. ^1H-^1H COSY spectrum (400 MHz, CD_3OD) of compound **4**.

Figure S26. HMBC spectrum (400 MHz, CD_3OD) of compound **4**.

Figure S27. NOESY spectrum of (400 MHz, CD3OD) compound **4.**

Figure S28. HR-ESI-MS of compound **4.**

Figure S29. 1H NMR spectrum (400 MHz, CD_3OD) of compound **5**.

Figure S30. ^{13}C NMR and DEPT spectra (100 MHz, CD_3OD) of compound **5**.

Figure S31. HMQC spectrum (400 MHz, CD_3OD) of compound **5.**

Figure S32. ^1H-^1H COSY spectrum (400 MHz, CD_3OD) of compound **5**.

Figure S33. HMBC spectrum (400 MHz, CD_3OD) of compound **5**.

Figure S34. HR-ESI-MS of compound **5.**

Figure S35. 1H NMR spectrum (400 MHz, CD_2Cl) of compound **6**.

Figure S36. ^{13}C NMR and DEPT spectra (100 MHz, CD_2Cl) of compound **6**.

Figure S37. HMQC spectrum (400 MHz, CD_3OD) of compound **6.**

Figure S38. ^1H-^1H COSY spectrum (400 MHz, CD_3OD) of compound **6**.

Figure S39. HMBC spectrum (400 MHz, CD_3OD) of compound **6**.

Figure S40. NOESY spectrum of (400 MHz, CD3OD) compound **6.**

Figure S41. HR-ESI-MS of compound **6.**

Figure S42. Calculated and experimental ECD spectra of compound **6.**

Figure S1. ^{1}H NMR spectrum (400 MHz, CD_3OD) of compound **1**.

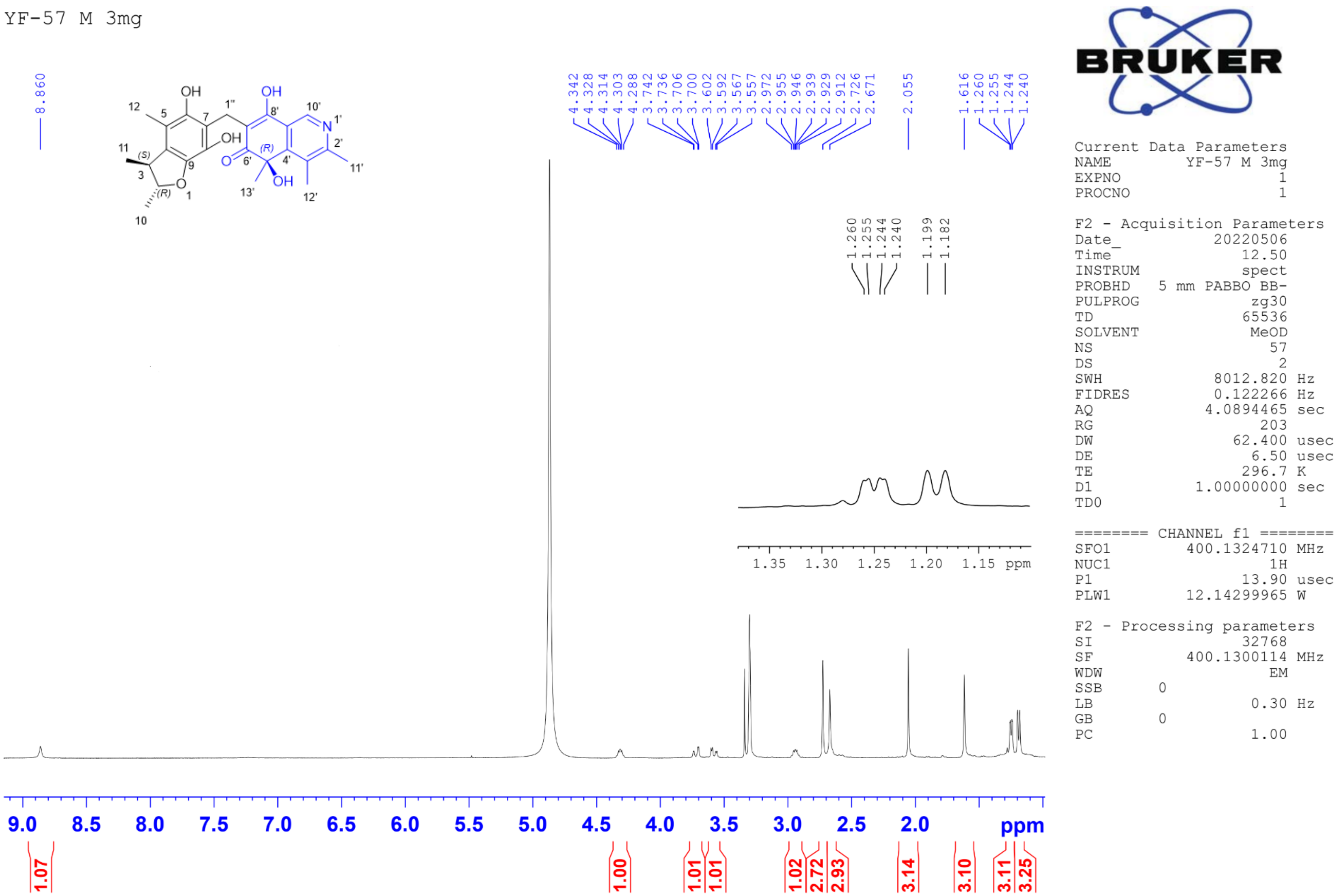

Figure S2. ^{13}C NMR and DEPT spectra (100 MHz, CD_3OD) of compound **1**.

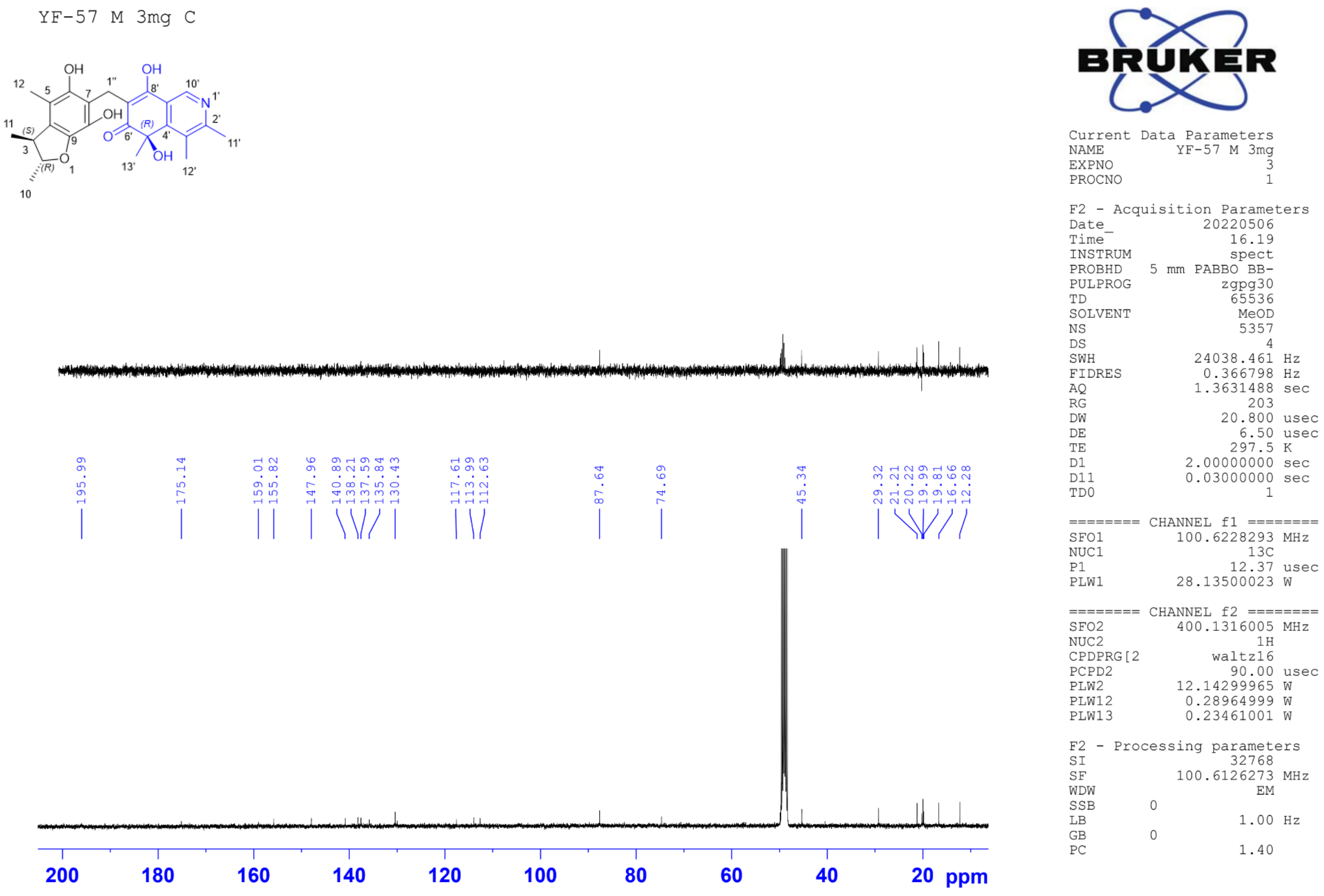

Figure S3. HMQC spectrum (400 MHz, CD_3OD) of compound **1**.

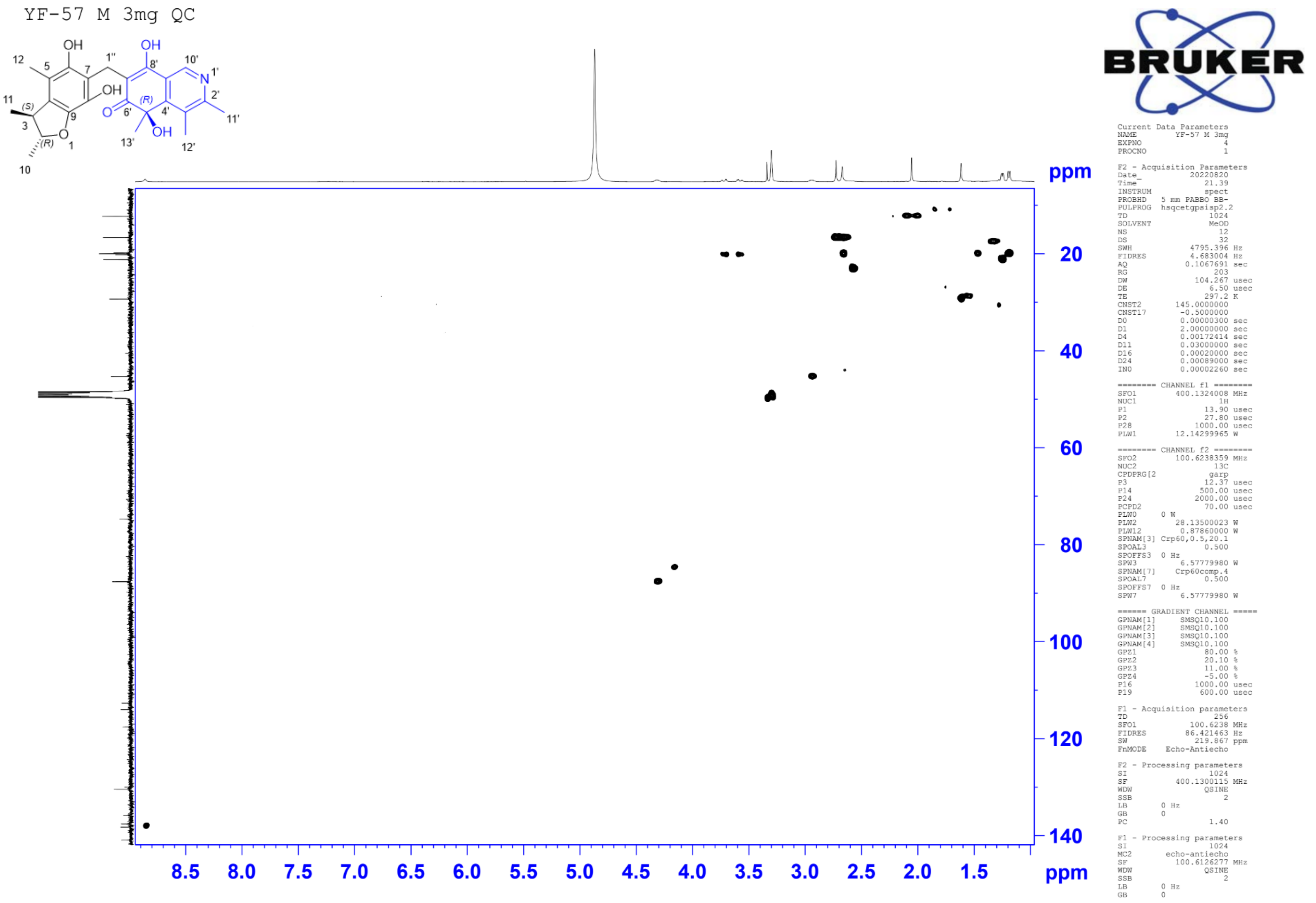

Figure S4. ^{1}H-^{1}H COSY spectrum (400 MHz, CD_3OD) of compound **1**.

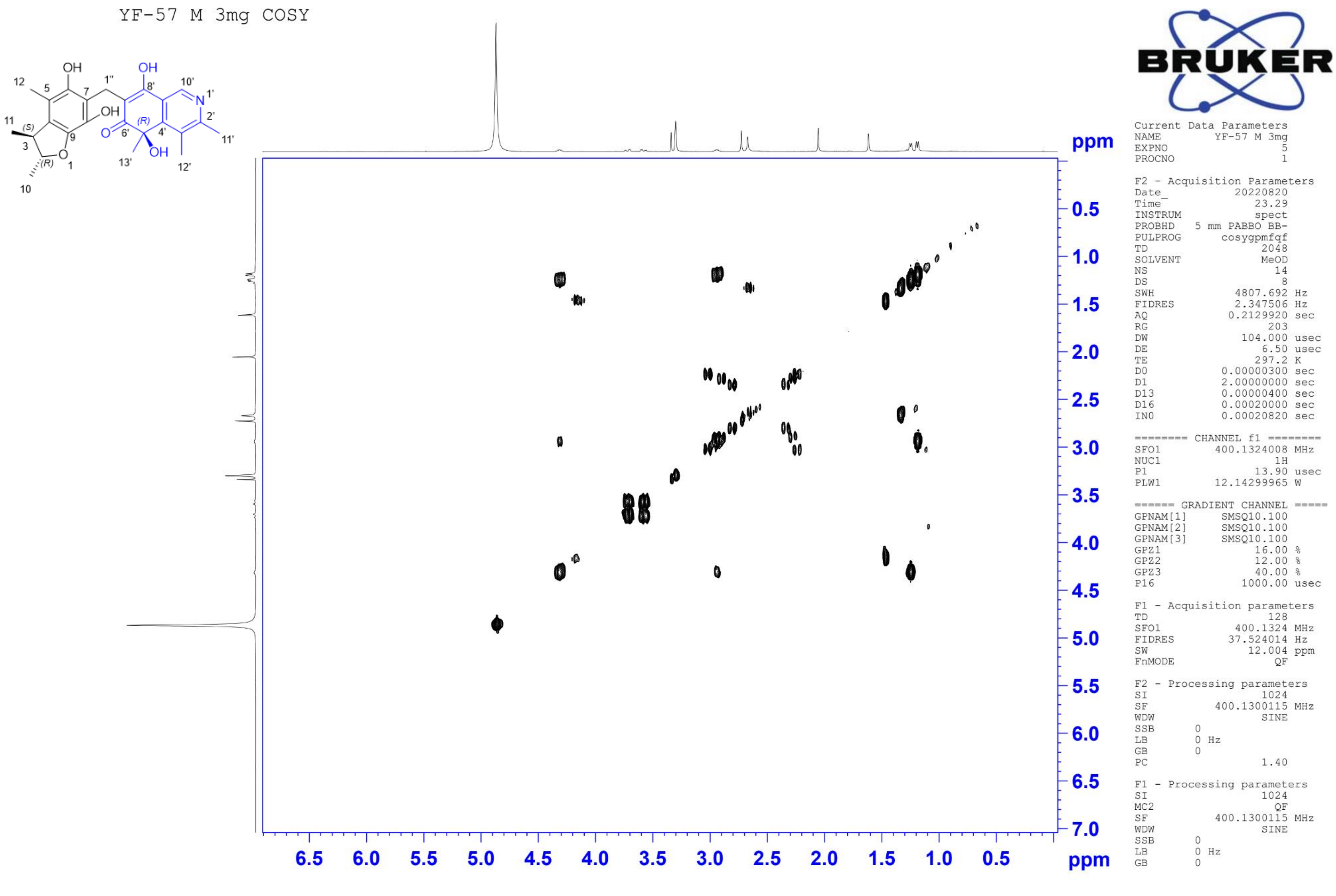

Figure S5. HMBC spectrum (400 MHz, CD_3OD) of compound **1**.

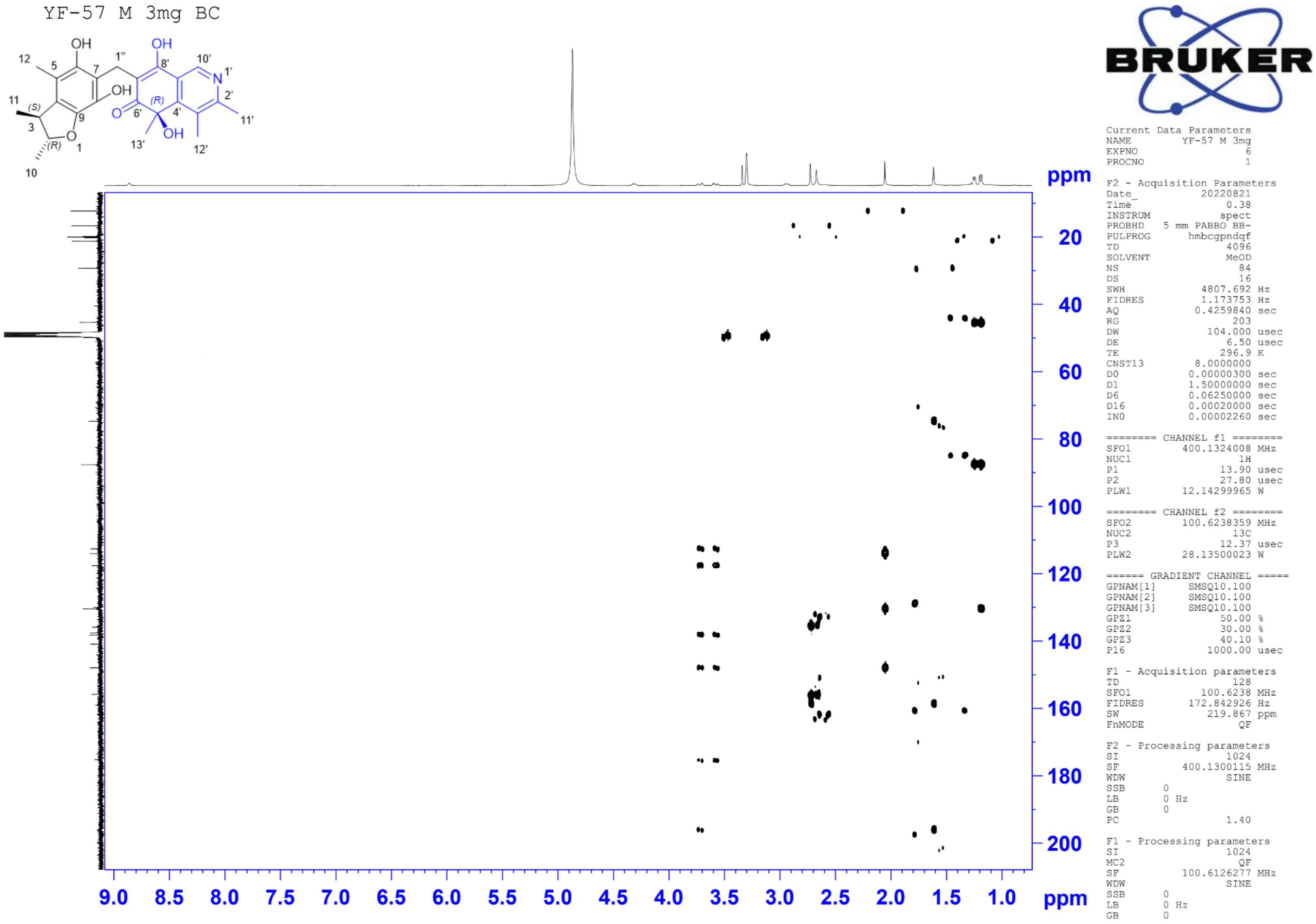

Figure S6. NOESY spectrum of (400 MHz, CD_3OD) compound **1**.

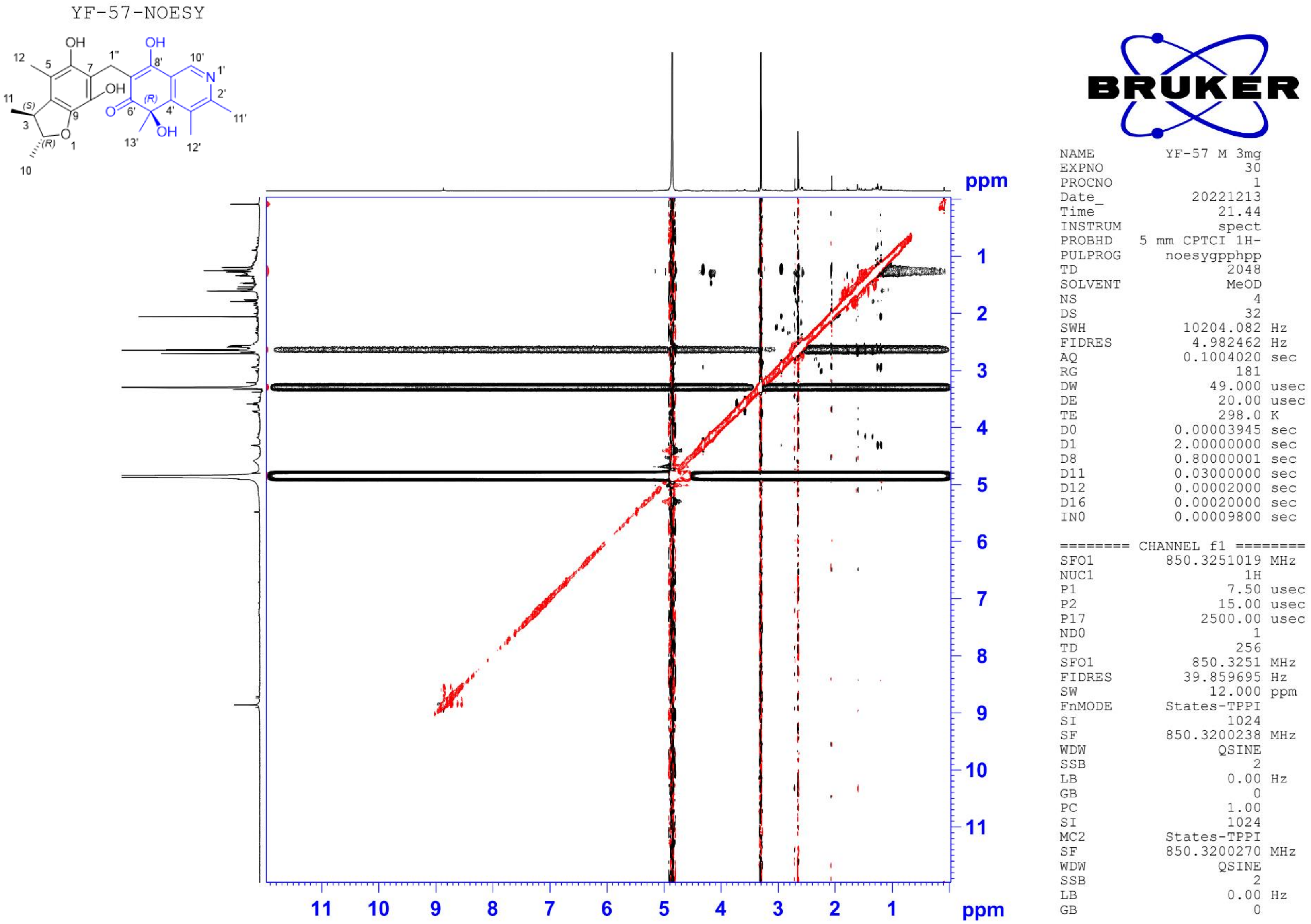

Figure S7. HR-ESI-MS of compound **1**.

Elemental Composition Report

Single Mass Analysis

Tolerance = 30.0 PPM / DBE: min = -1.5, max = 50.0
Element prediction: Off
Number of isotope peaks used for i-FIT = 3

Monoisotopic Mass, Even Electron Ions
209 formula(e) evaluated with 7 results within limits (up to 50 closest results for each mass)
Elements Used:
C: 0-30 H: 0-50 N: 0-5 O: 0-8
YF-57-N 74 (0.304) Cm (74)
1: TOF MS ES-

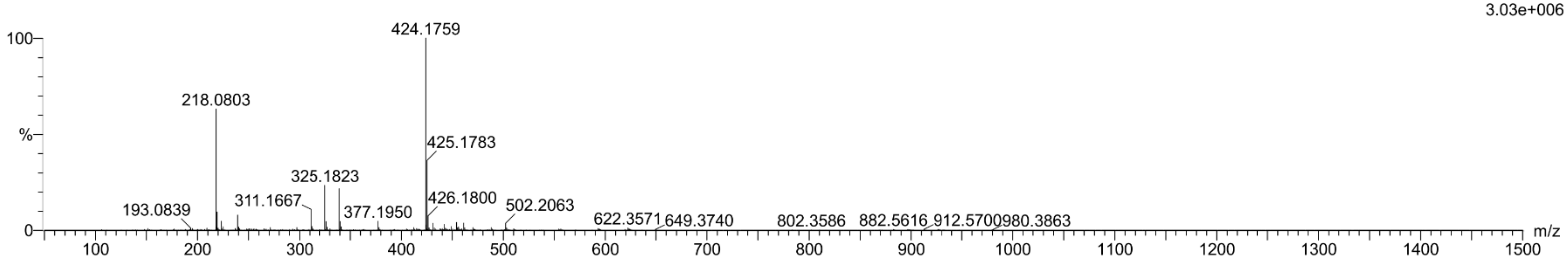

Minimum:				-1.5				
Maximum:		5.0	30.0	50.0				
Mass	Calc. Mass	mDa	PPM	DBE	i-FIT	Norm	Conf(%)	Formula
424.1759	424.1760	-0.1	-0.2	12.5	937.2	2.911	5.44	C24 H26 N O6
	424.1773	-1.4	-3.3	17.5	936.3	2.019	13.28	C25 H22 N5 O2
	424.1720	3.9	9.2	8.5	937.5	3.152	4.28	C19 H26 N3 O8
	424.1814	-5.5	-13.0	21.5	934.8	0.436	64.67	C30 H22 N3
	424.1832	-7.3	-17.2	8.5	937.8	3.480	3.08	C18 H26 N5 O7
	424.1661	9.8	23.1	17.5	937.0	2.660	7.00	C26 H22 N3 O3
	424.1872	-11.3	-26.6	12.5	938.1	3.793	2.25	C23 H26 N3 O5

Figure S8. ^{1}H NMR spectrum (400 MHz, CD_3OD) of compound **2**.

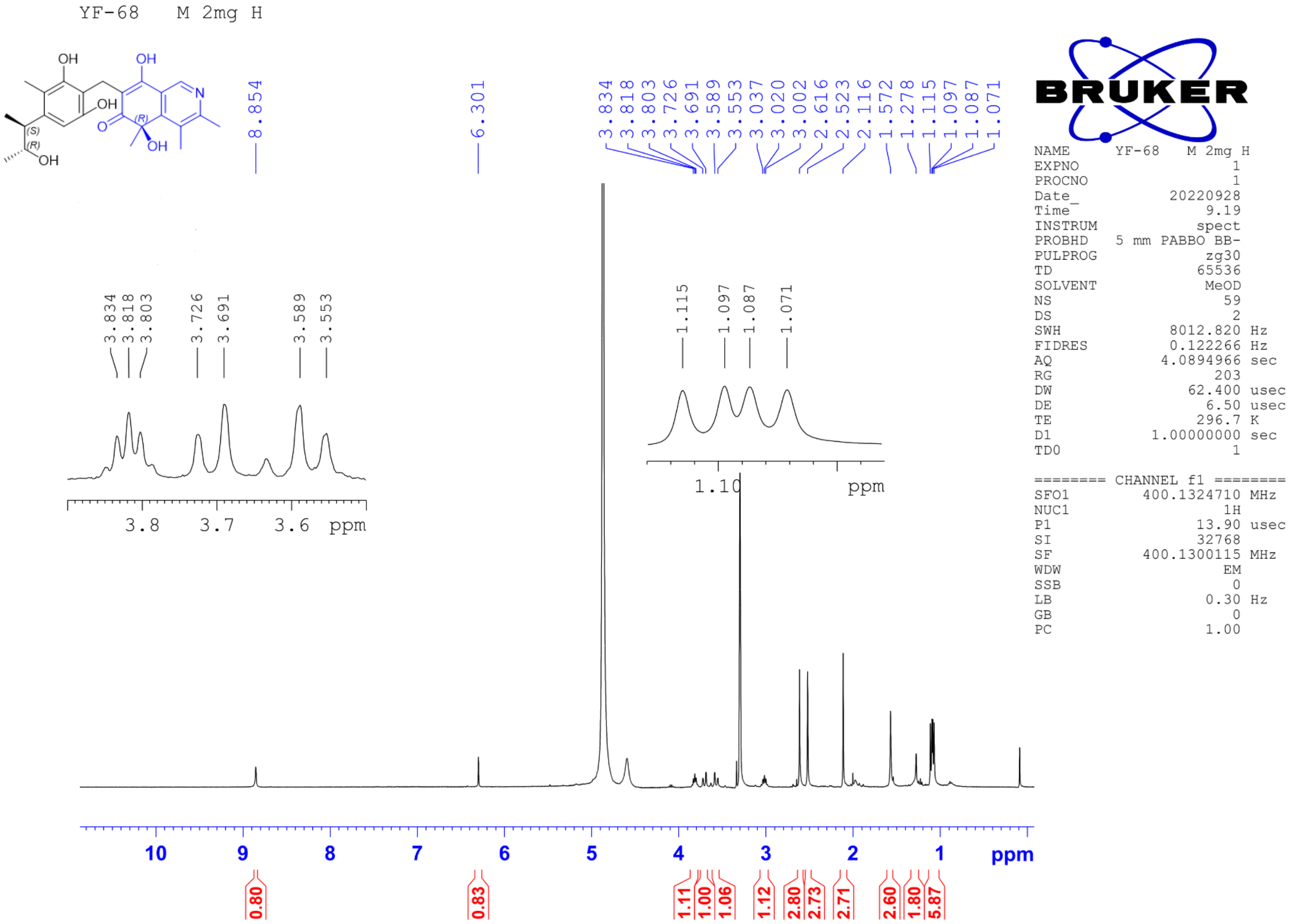

Figure S9. ^{13}C NMR and DEPT spectra (100 MHz, CD_3OD) of compound **2**.

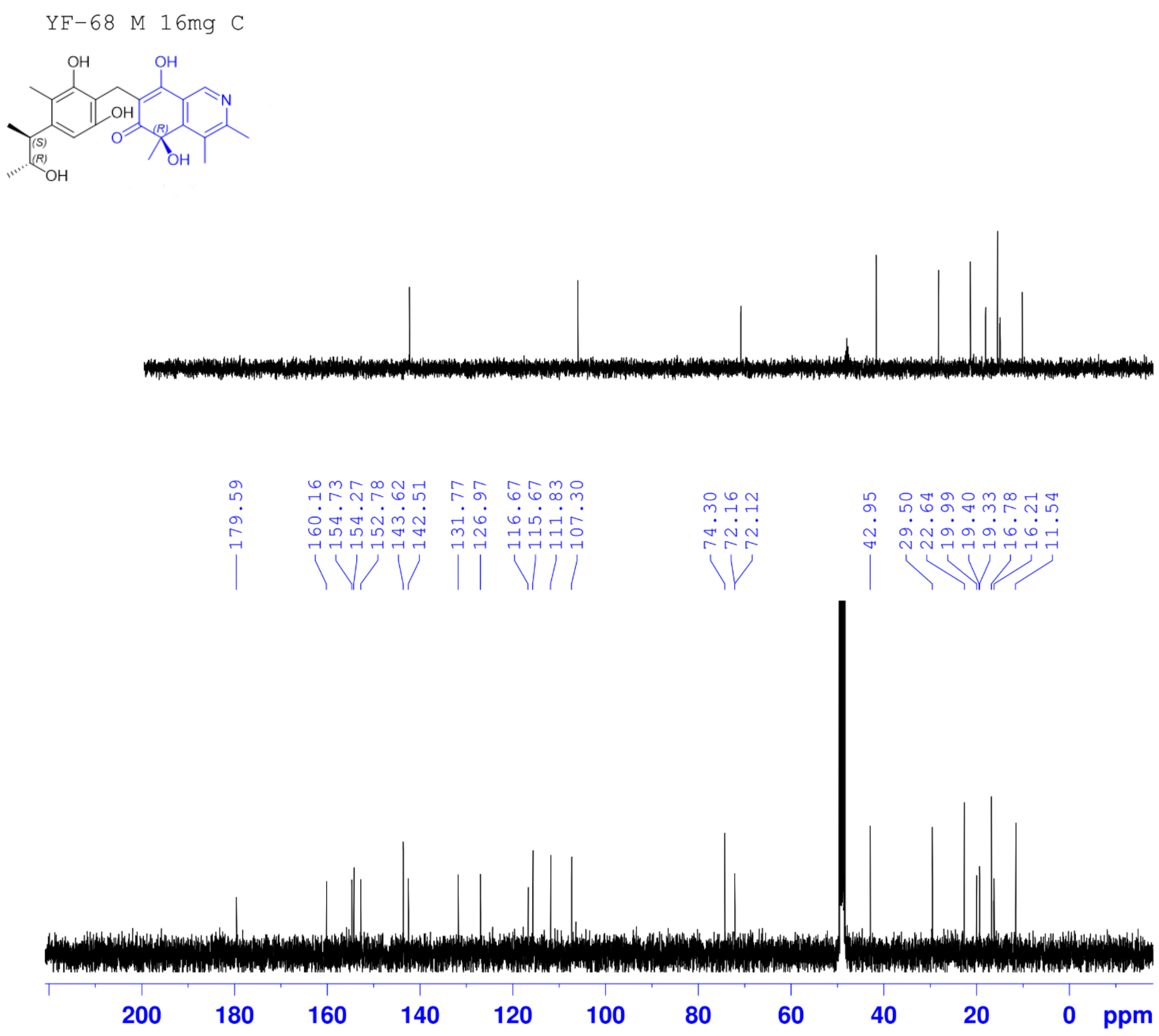

NAME	YF-68 M 16mg
EXPNO	2
PROCNO	1
Date_	20220520
Time	20.33
INSTRUM	spect
PROBHD	5 mm PABBO BB-
PULPROG	zgpg30
TD	65536
SOLVENT	MeOD
NS	212
DS	4
SWH	24038.461 Hz
FIDRES	0.366798 Hz
AQ	1.3631988 sec
RG	203
DW	20.800 usec
DE	6.50 usec
TE	296.7 K
D1	2.00000000 sec
D11	0.03000000 sec
TD0	1
======== CHANNEL f1 ========	
SFO1	100.6228293 MHz
NUC1	13C
P1	12.37 usec
SI	32768
SF	100.6126295 MHz
WDW	EM
SSB	0
LB	1.00 Hz
GB	0
PC	1.40

Figure S10. HMQC spectrum (400 MHz, CD_3OD) of compound **2**.

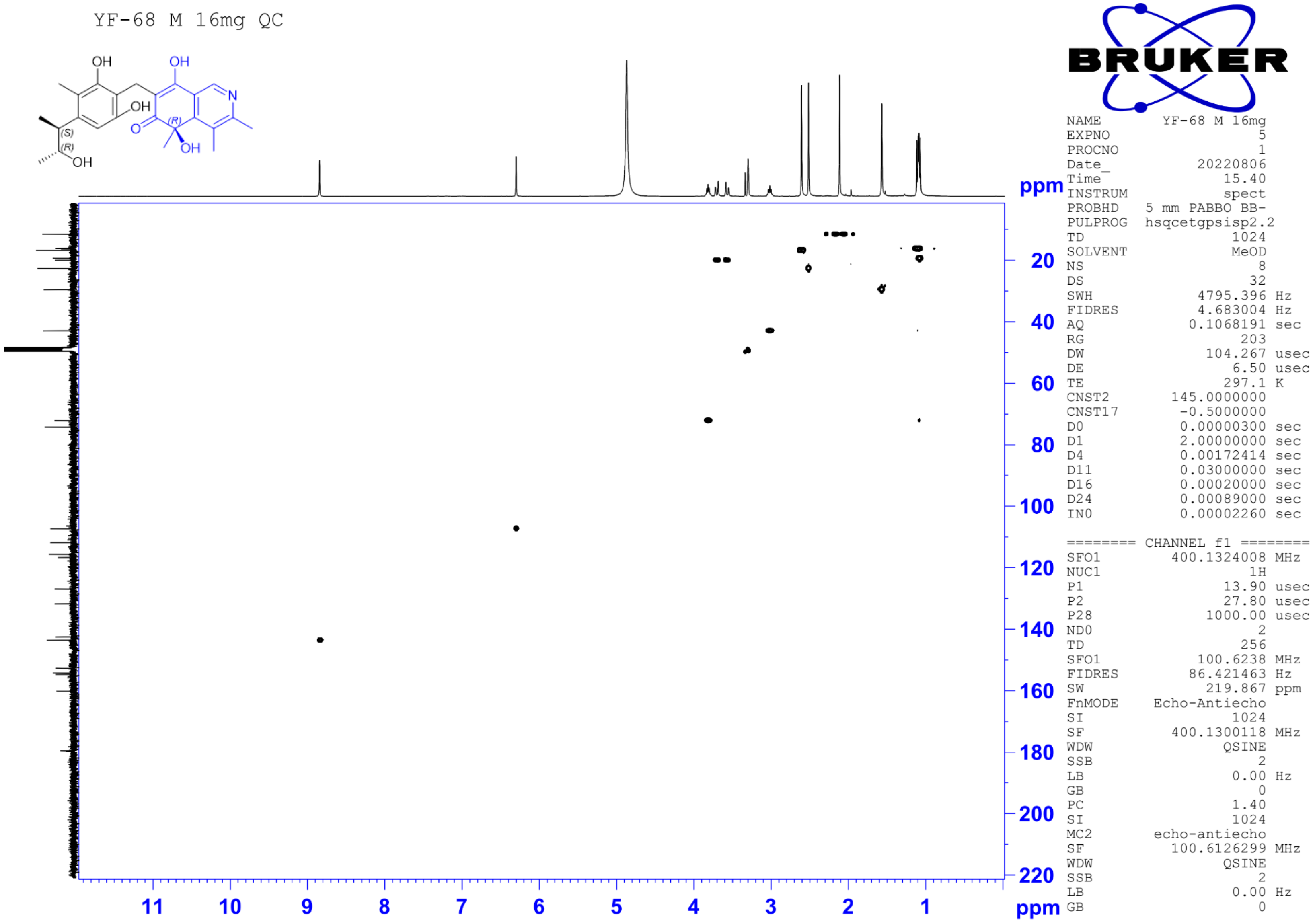

Figure S11. ^{1}H-^{1}H COSY spectrum (400 MHz, CD_3OD) of compound **2**.

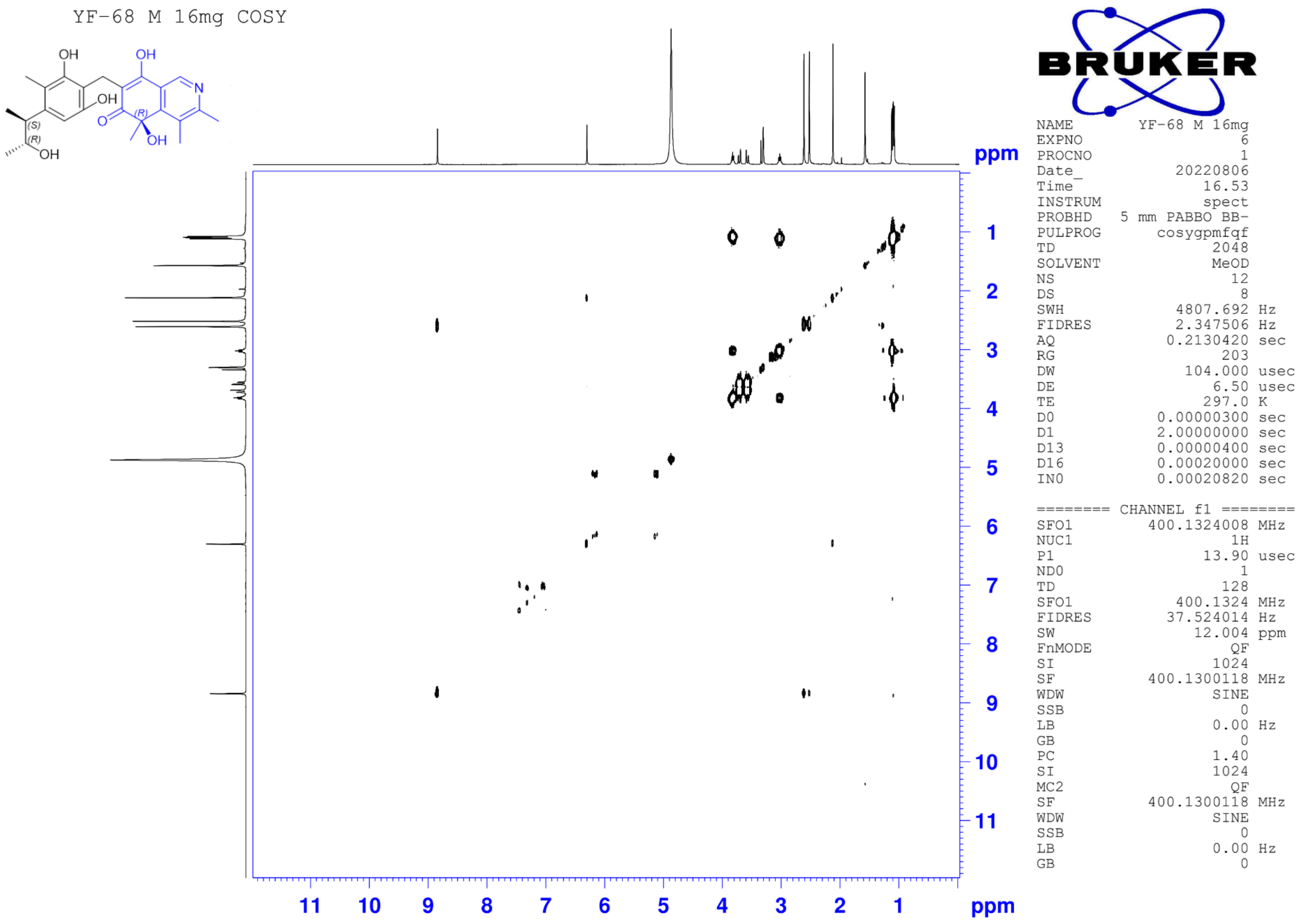

Figure S12. HMBC spectrum (400 MHz, CD_3OD) of compound **2**.

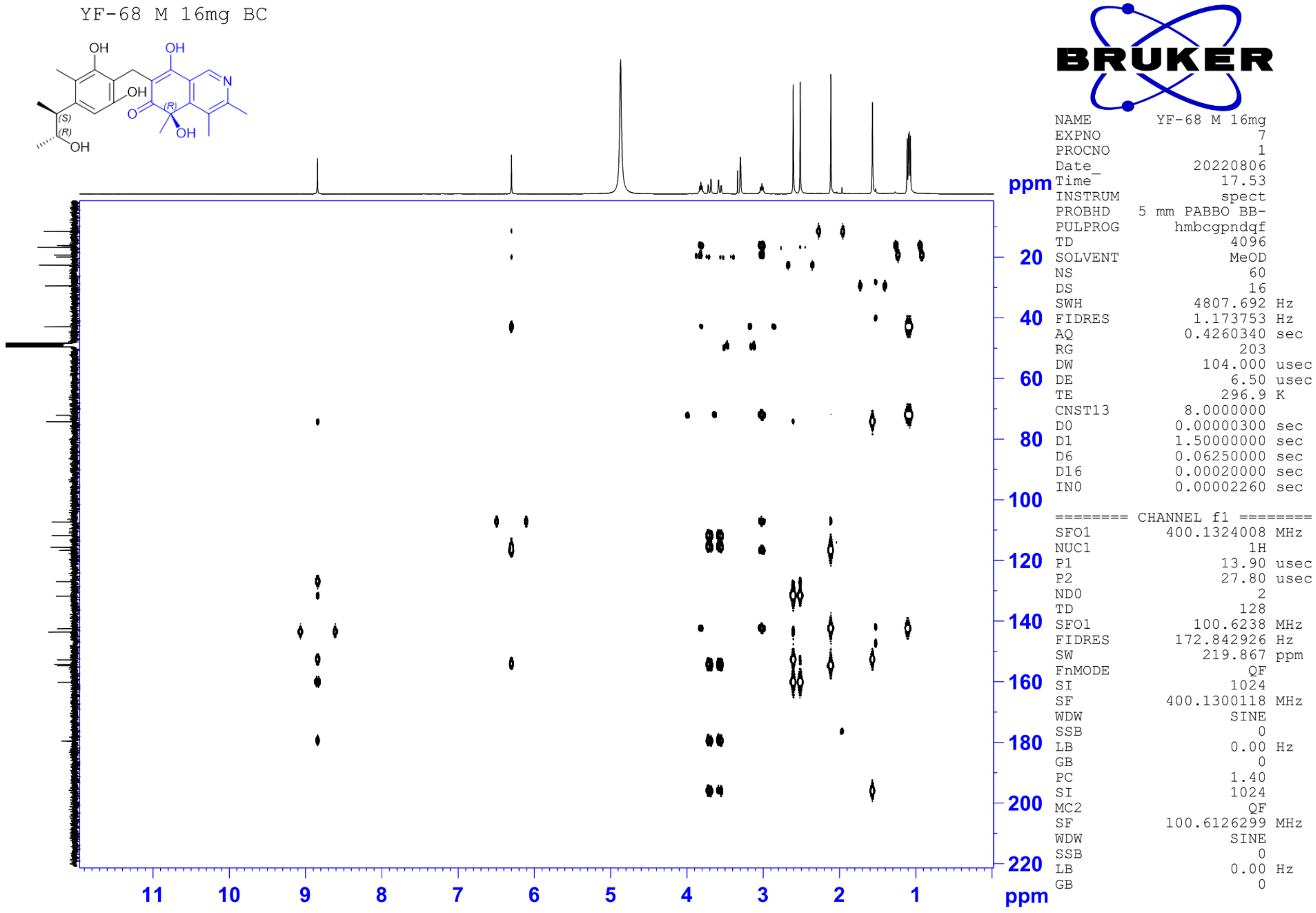

Figure S13. NOESY spectrum of (400 MHz, CD_3OD) compound **2**.

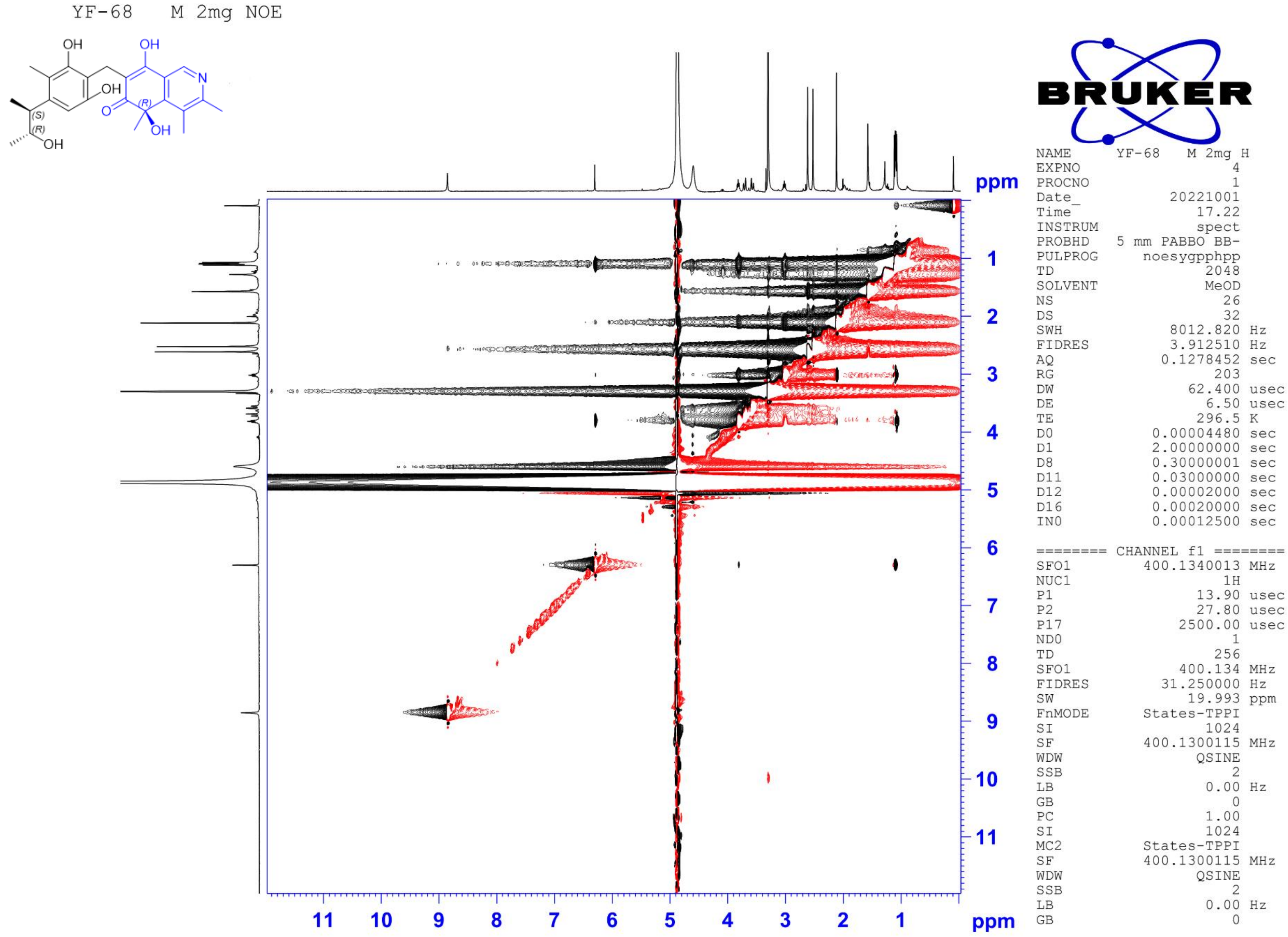

Figure S14. HR-ESI-MS of compound **2**.

Elemental Composition Report

Single Mass Analysis

Tolerance = 30.0 PPM / DBE: min = -1.5, max = 50.0
Element prediction: Off
Number of isotope peaks used for i-FIT = 3

Monoisotopic Mass, Even Electron Ions
207 formula(e) evaluated with 7 results within limits (up to 50 closest results for each mass)
Elements Used:
C: 0-30 H: 0-50 N: 0-5 O: 0-8
YF-68-N 85 (0.343) Cm (85:86)
1: TOF MS ES-

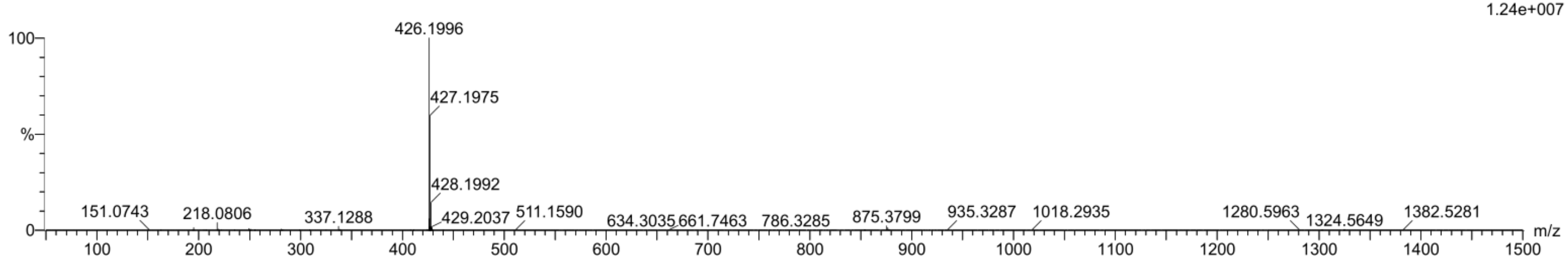

Minimum:				-1.5				
Maximum:		5.0	30.0	50.0				

Mass	Calc. Mass	mDa	PPM	DBE	i-FIT	Norm	Conf(%)	Formula
426.1996	426.1989	0.7	1.6	7.5	1101.2	2.302	10.01	C18 H28 N5 O7
	426.1970	2.6	6.1	20.5	1100.3	1.366	25.52	C30 H24 N3
	426.2029	-3.3	-7.7	11.5	1101.1	2.133	11.85	C23 H28 N3 O5
	426.1930	6.6	15.5	16.5	1100.7	1.799	16.54	C25 H24 N5 O2
	426.2069	-7.3	-17.1	15.5	1100.7	1.809	16.38	C28 H28 N O3
	426.1917	7.9	18.5	11.5	1101.1	2.191	11.18	C24 H28 N O6
	426.1876	12.0	28.2	7.5	1101.4	2.463	8.52	C19 H28 N3 O8

Figure S15. ^{1}H NMR spectrum (400 MHz, CD_3OD) of compound **3**.

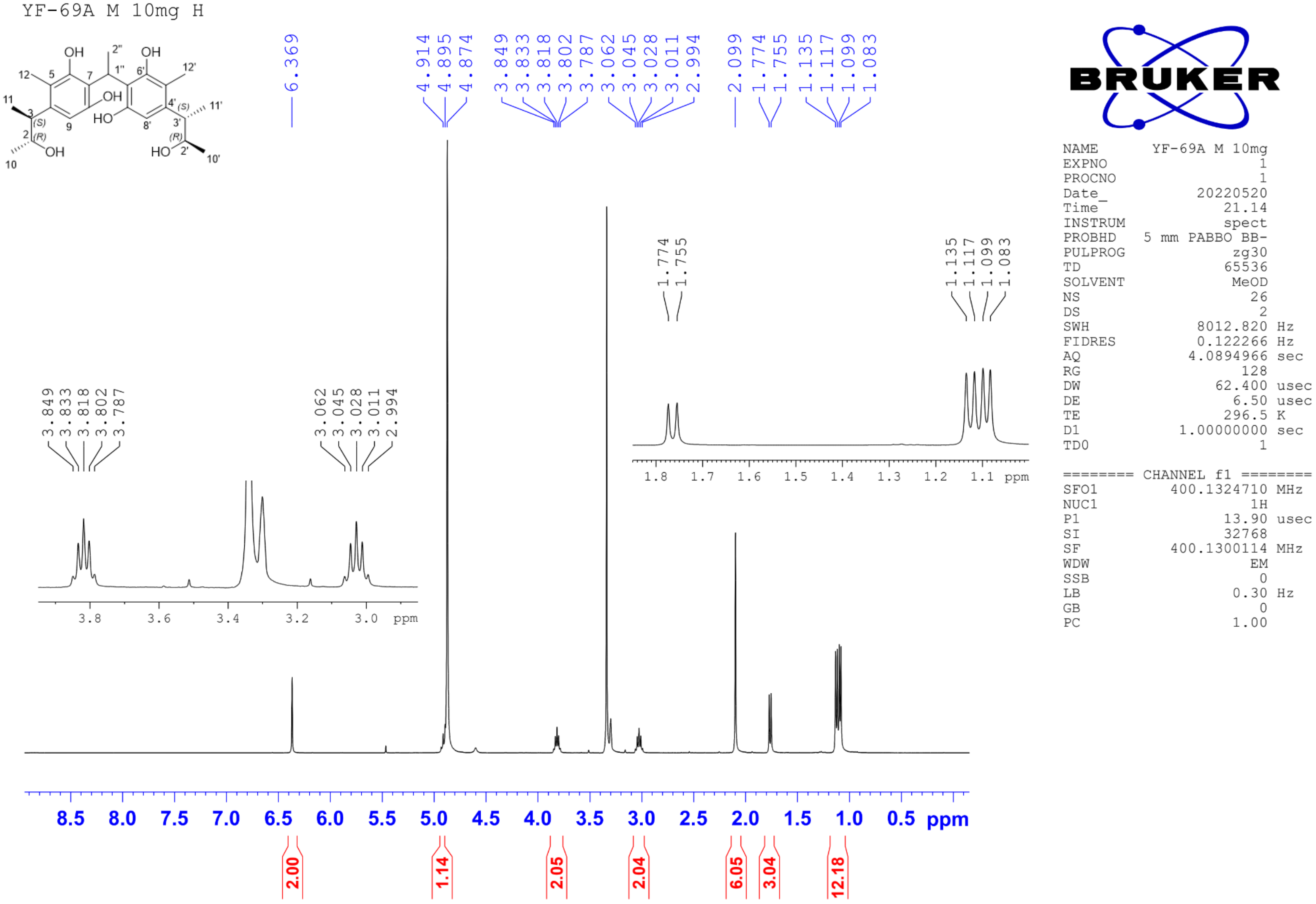

Figure S16. ^{13}C NMR and DEPT spectra (100 MHz, CD_3OD) of compound **3**.

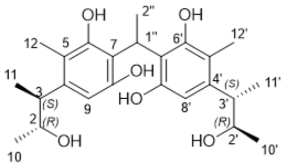

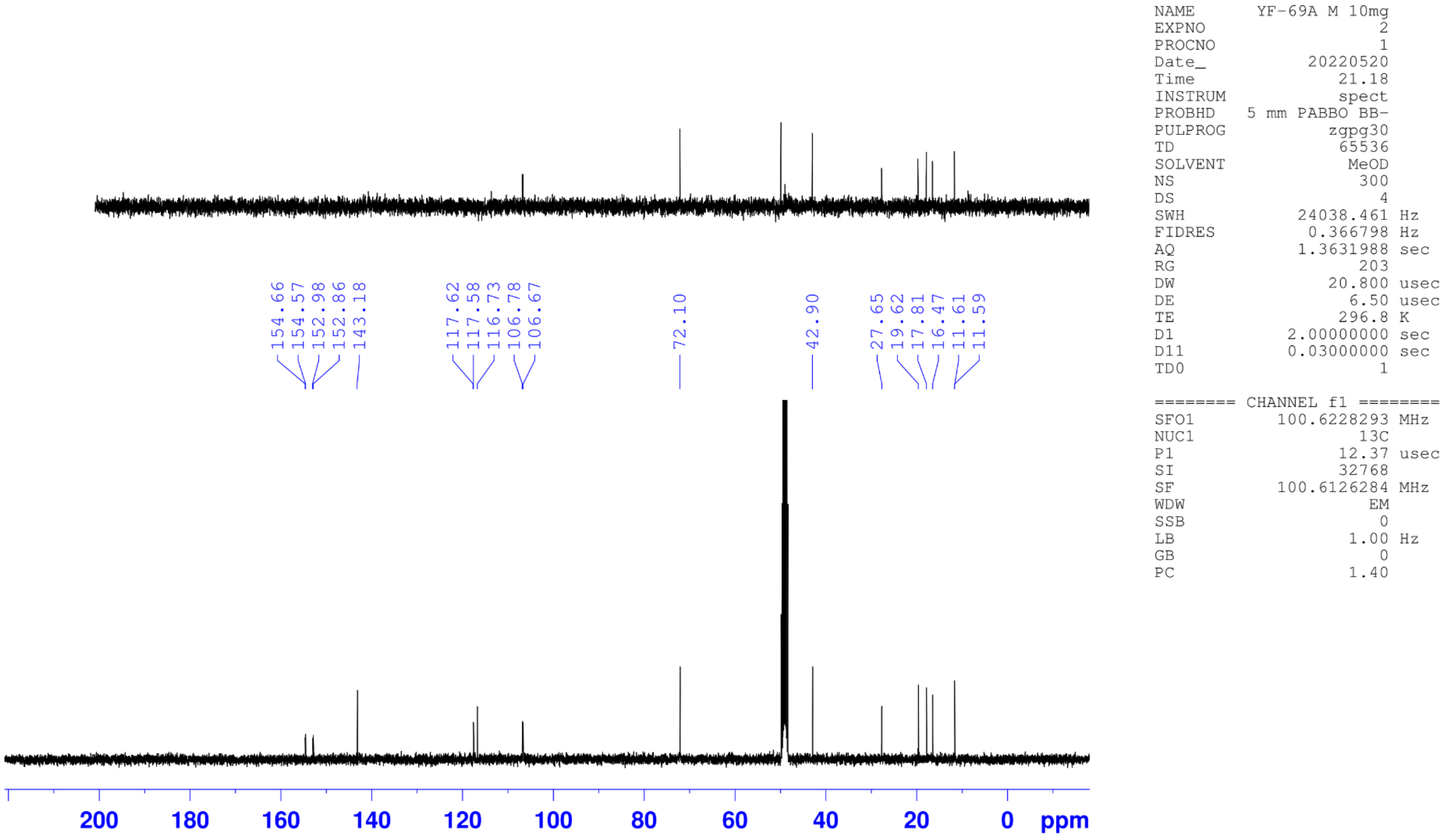

Figure S17. HMQC spectrum (400 MHz, CD_3OD) of compound **3**.

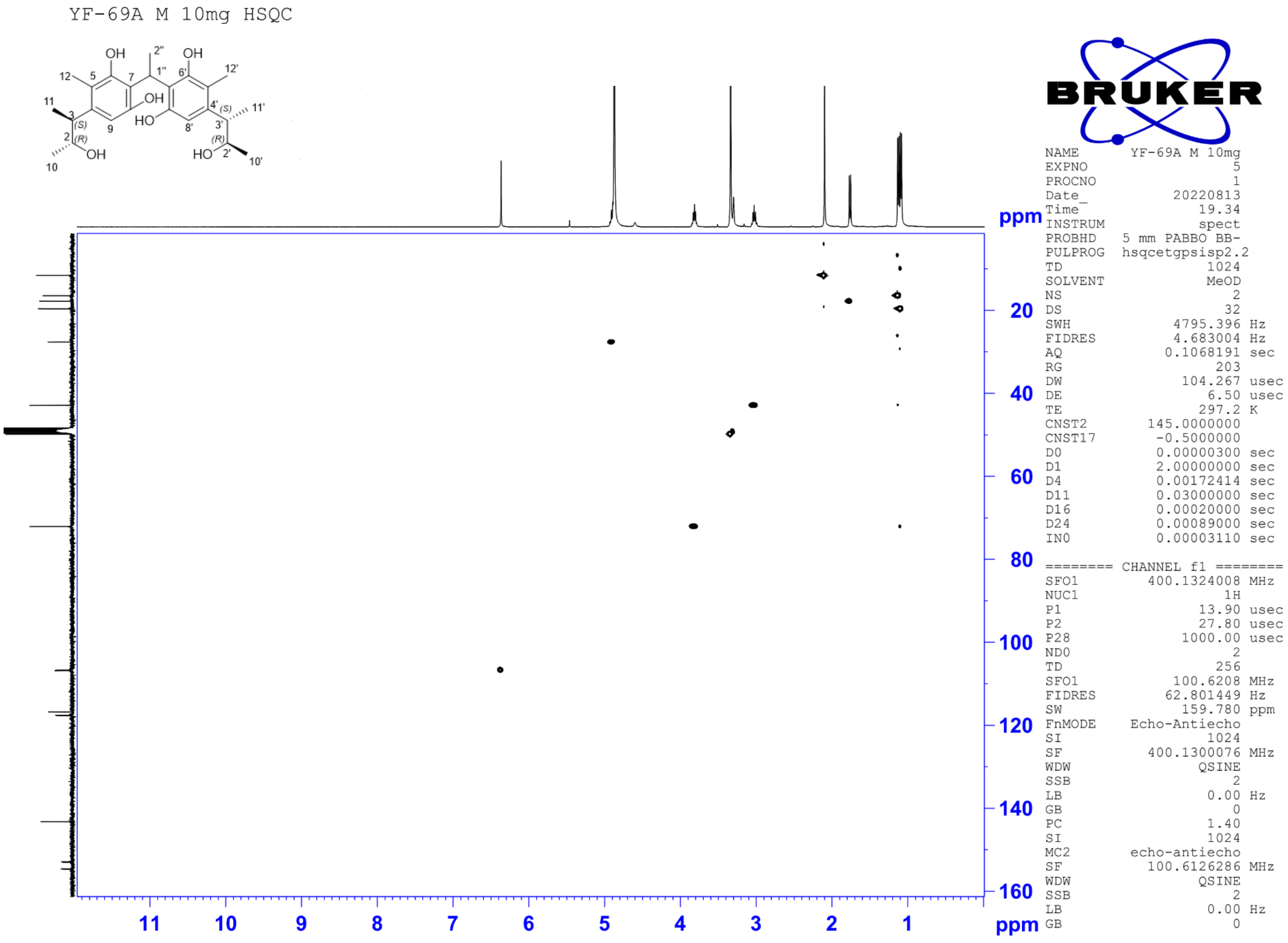

Figure S18. ^1H-^1H COSY spectrum (400 MHz, CD_3OD) of compound **3**.

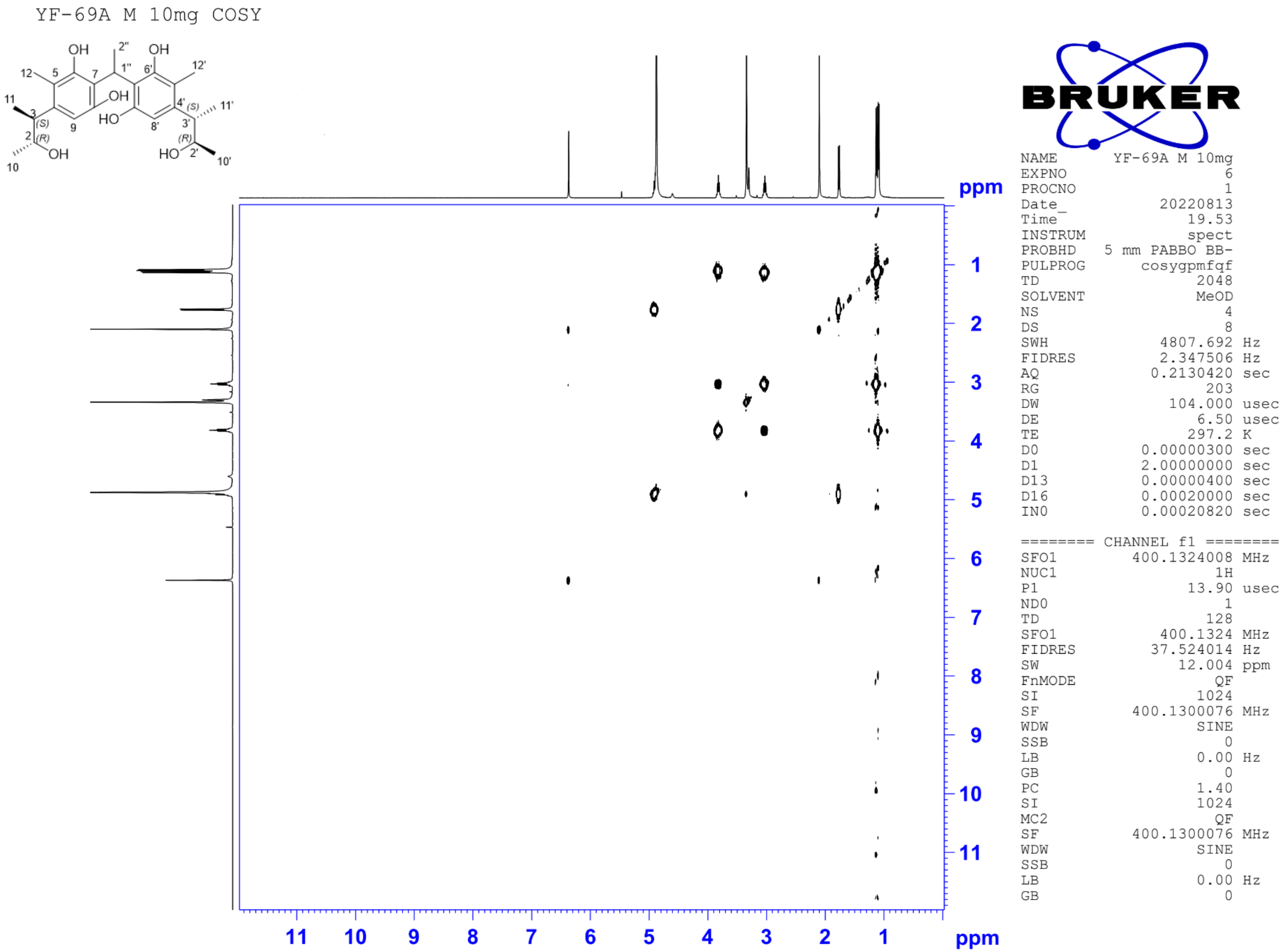

Figure S19. HMBC spectrum (400 MHz, CD_3OD) of compound **3**.

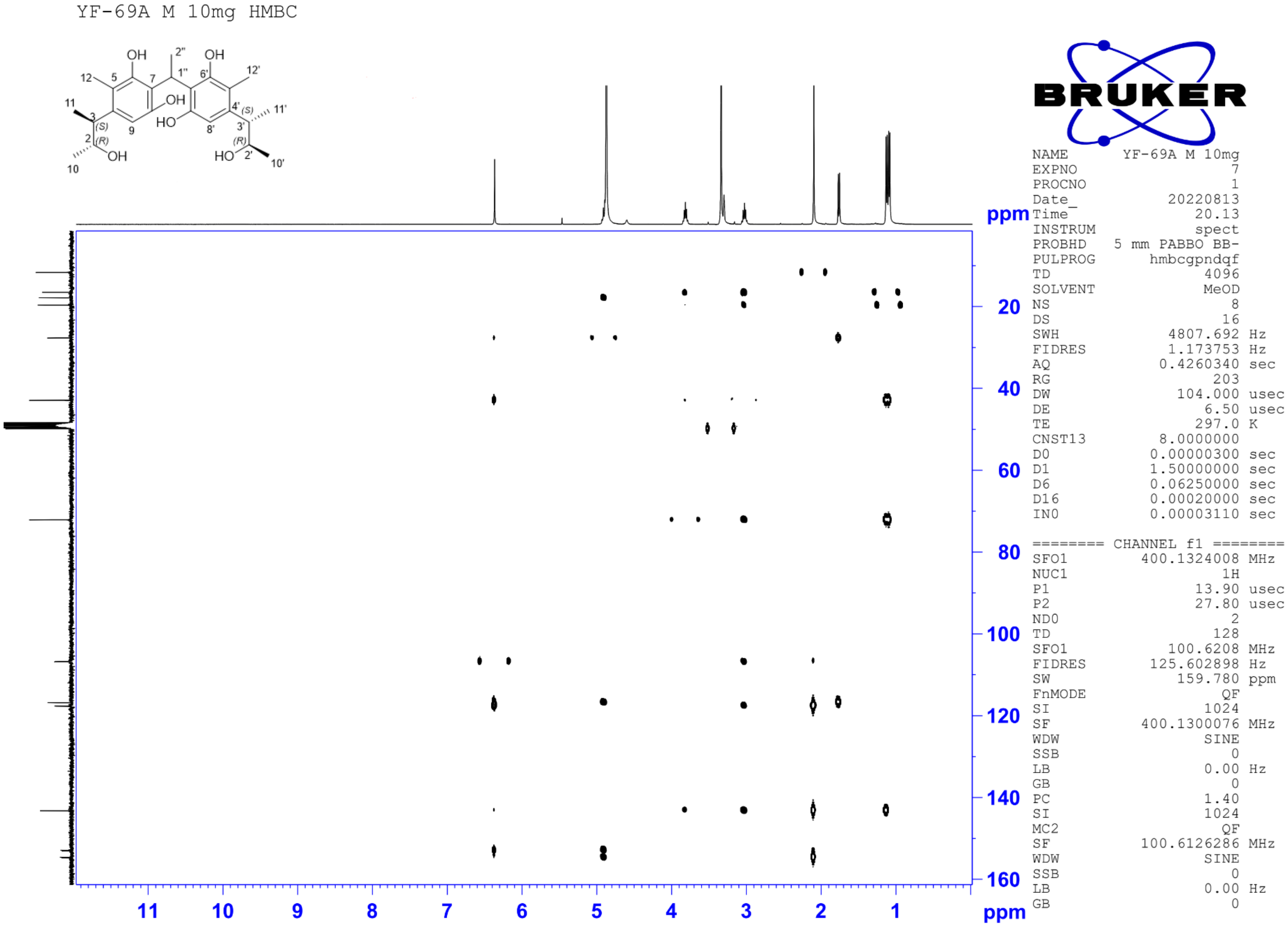

Figure S20. NOESY spectrum of (400 MHz, CD_3OD) compound **3**.

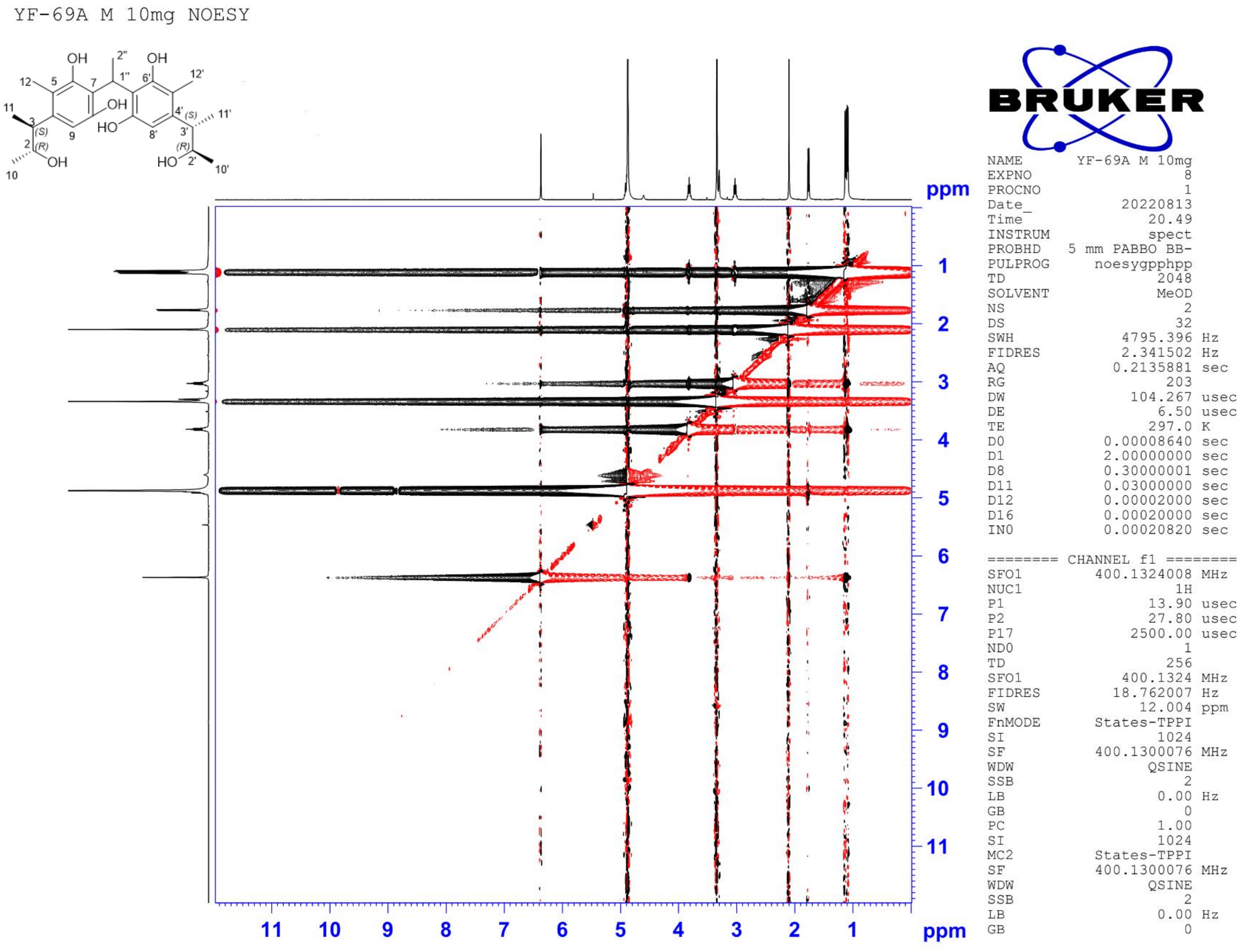

Figure S21. HR-ESI-MS of compound **3**.

Elemental Composition Report

Tolerance = 50.0 PPM / DBE: min = -1.5, max = 50.0
Element prediction: Off
Number of isotope peaks used for i-FIT = 3

Monoisotopic Mass, Even Electron Ions
103 formula(e) evaluated with 11 results within limits (up to 50 best isotopic matches for each mass)
Elements Used:
C: 5-35 H: 0-60 O: 0-10 23Na: 0-1
YF-69A 74 (0.304) Cm (72:77)
1: TOF MS ES+

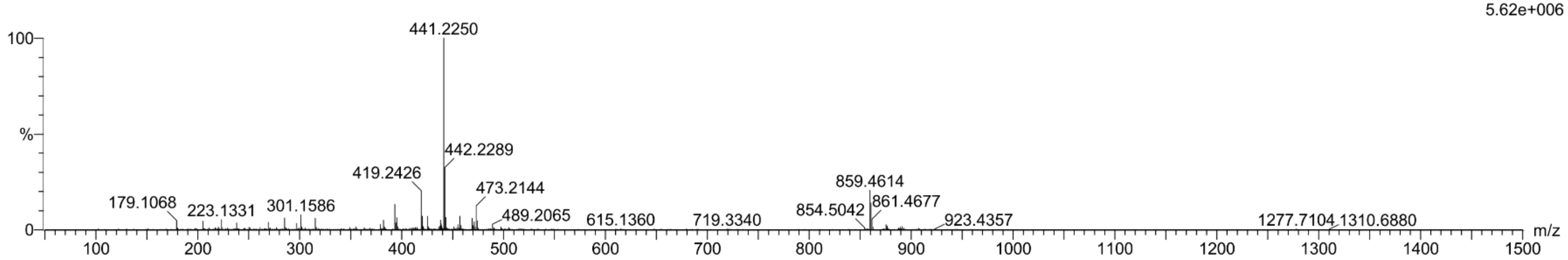

Minimum:	80.00				-1.5				
Maximum:	100.00		50.0	50.0	50.0				
Mass	RA	Calc. Mass	mDa	PPM	DBE	i-FIT	Norm	Conf(%)	Formula
441.2250	100.00	441.2194	5.6	12.7	16.5	1497.4	0.913	40.14	C31 H30 O 23Na
		441.2430	-18.0	-40.8	14.5	1497.7	1.229	29.25	C30 H33 O3
		441.2066	18.4	41.7	15.5	1498.1	1.611	19.96	C29 H29 O4
		441.2218	3.2	7.3	19.5	1499.1	2.618	7.29	C33 H29 O
		441.2406	-15.6	-35.4	11.5	1500.4	3.966	1.89	C28 H34 O3 23Na
		441.2277	-2.7	-6.1	10.5	1501.1	4.623	0.98	C26 H33 O6
		441.2042	20.8	47.1	12.5	1502.2	5.722	0.33	C27 H30 O4 23Na
		441.2253	-0.3	-0.7	7.5	1503.1	6.637	0.13	C24 H34 O6 23Na
		441.2125	12.5	28.3	6.5	1505.5	9.027	0.01	C22 H33 O9
		441.2101	14.9	33.8	3.5	1506.9	10.454	0.00	C20 H34 O9 23Na
		441.2464	-21.4	-48.5	2.5	1507.0	10.472	0.00	C21 H38 O8 23Na

Figure S22. ^{1}H NMR spectrum (400 MHz, CD_3OD) of compound **4**.

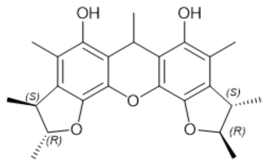

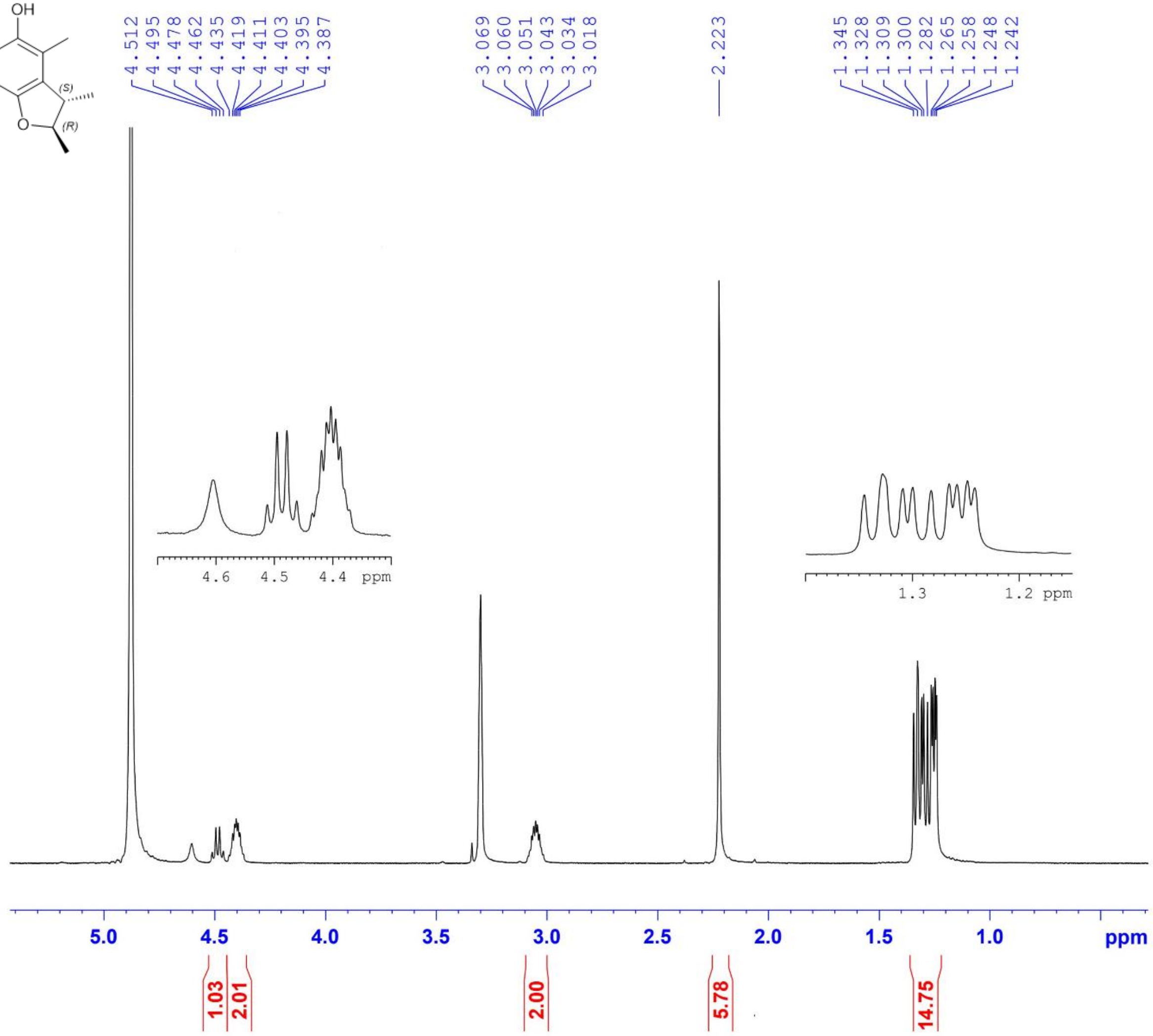


```
NAME          YF-115 M 3mg
EXPNO                    1
PROCNO                   1
Date_             20220708
Time                 20.27
INSTRUM              spect
PROBHD   5 mm PABBO BB-
PULPROG               zg30
TD                   65536
SOLVENT               MeOD
NS                      79
DS                       2
SWH               8012.820 Hz
FIDRES            0.122266 Hz
AQ               4.0894966 sec
RG                     203
DW                  62.400 usec
DE                    6.50 usec
TE                   295.5 K
D1              1.00000000 sec
TD0                      1

======== CHANNEL f1 ========
SFO1           400.1324710 MHz
NUC1                    1H
P1                   13.90 usec
SI                   32768
SF             400.1300114 MHz
WDW                     EM
SSB                      0
LB                    0.30 Hz
GB                       0
PC                    1.00
```

Figure S23. ^{13}C NMR and DEPT spectra (100 MHz, CD_3OD) of compound **4**.

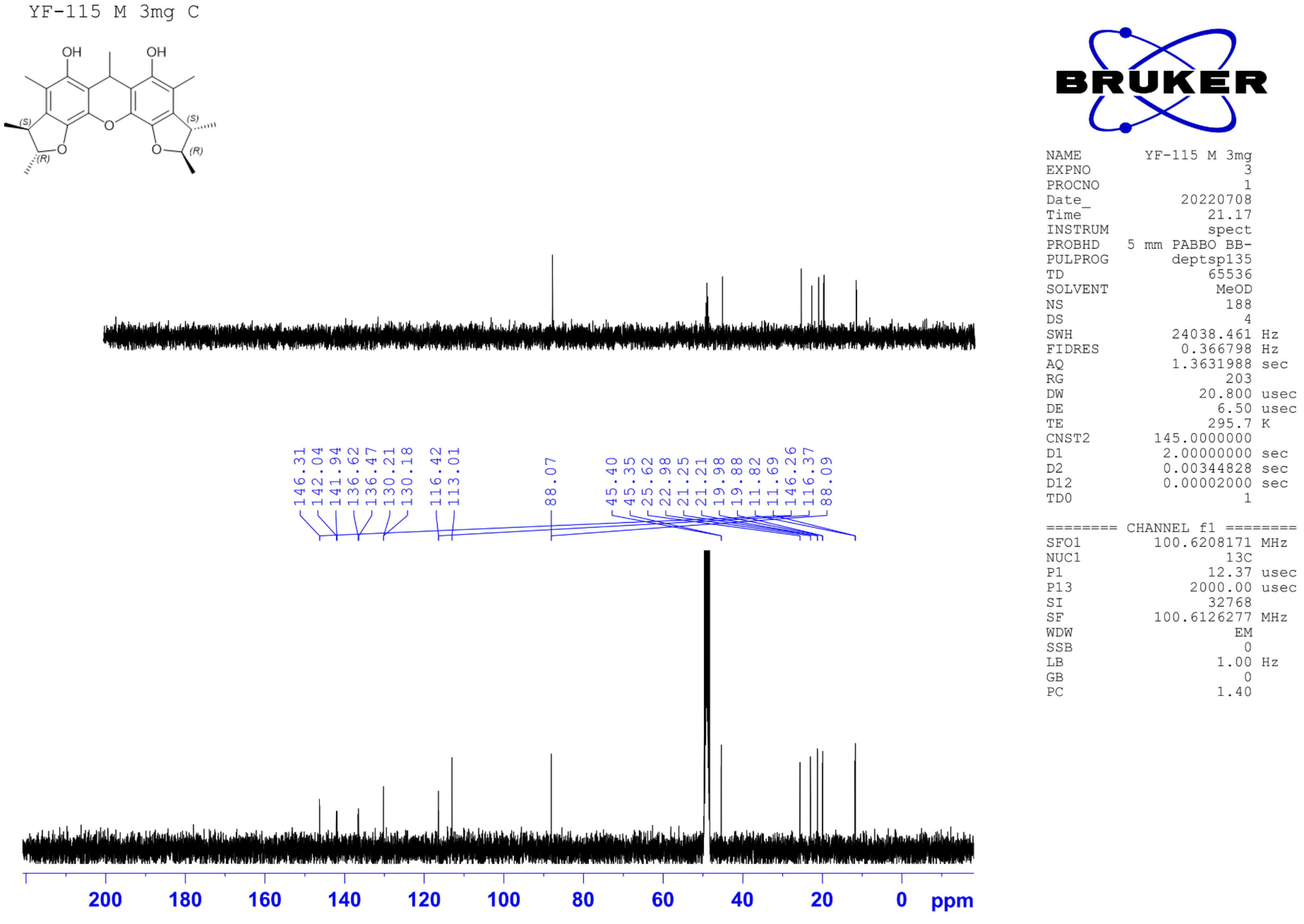

Figure S24. HMQC spectrum (400 MHz, CD_3OD) of compound **4**.

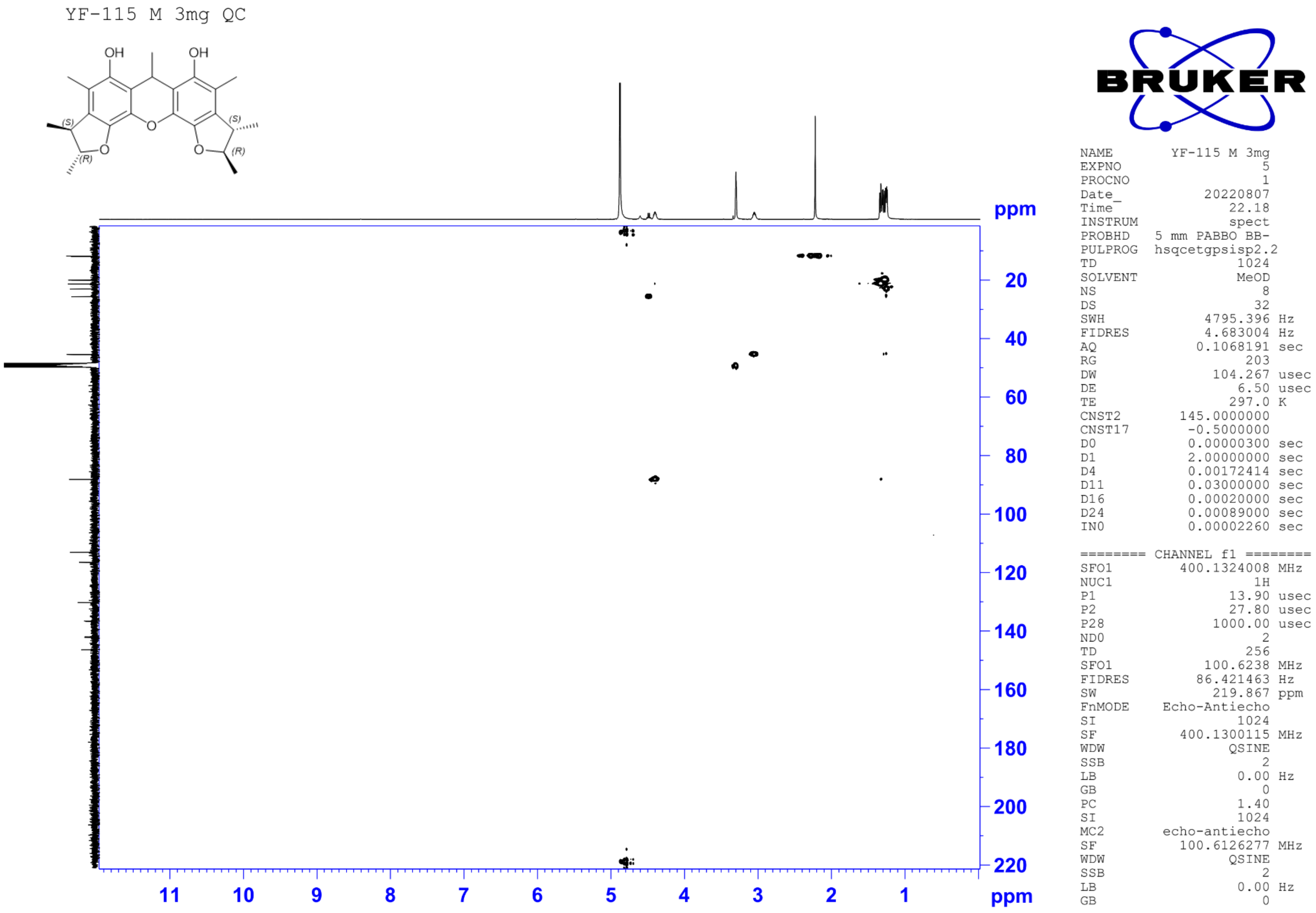

Figure S25. ^{1}H-^{1}H COSY spectrum (400 MHz, CD_3OD) of compound **4**.

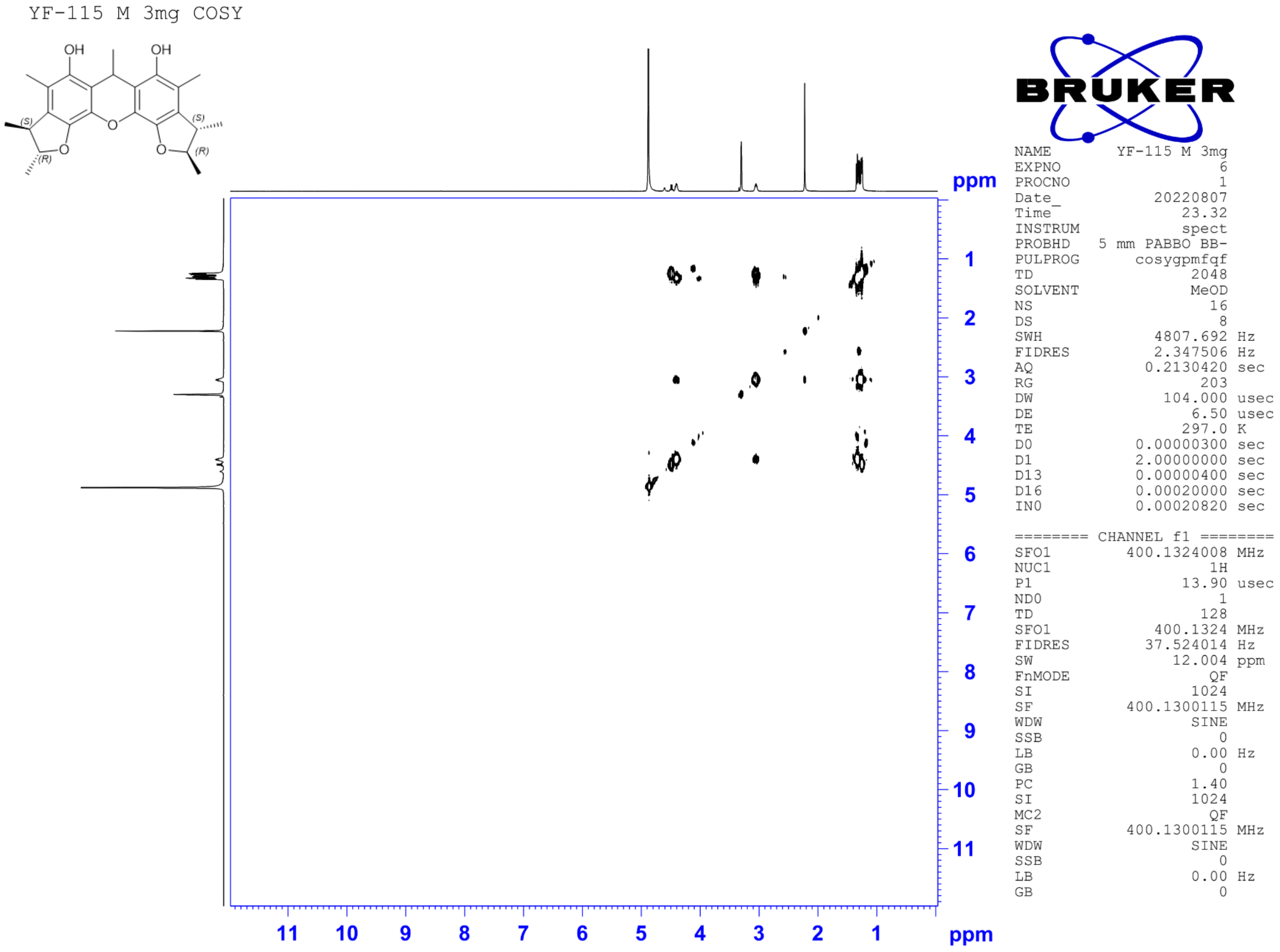

Figure S26. HMBC spectrum (400 MHz, CD_3OD) of compound **4**.

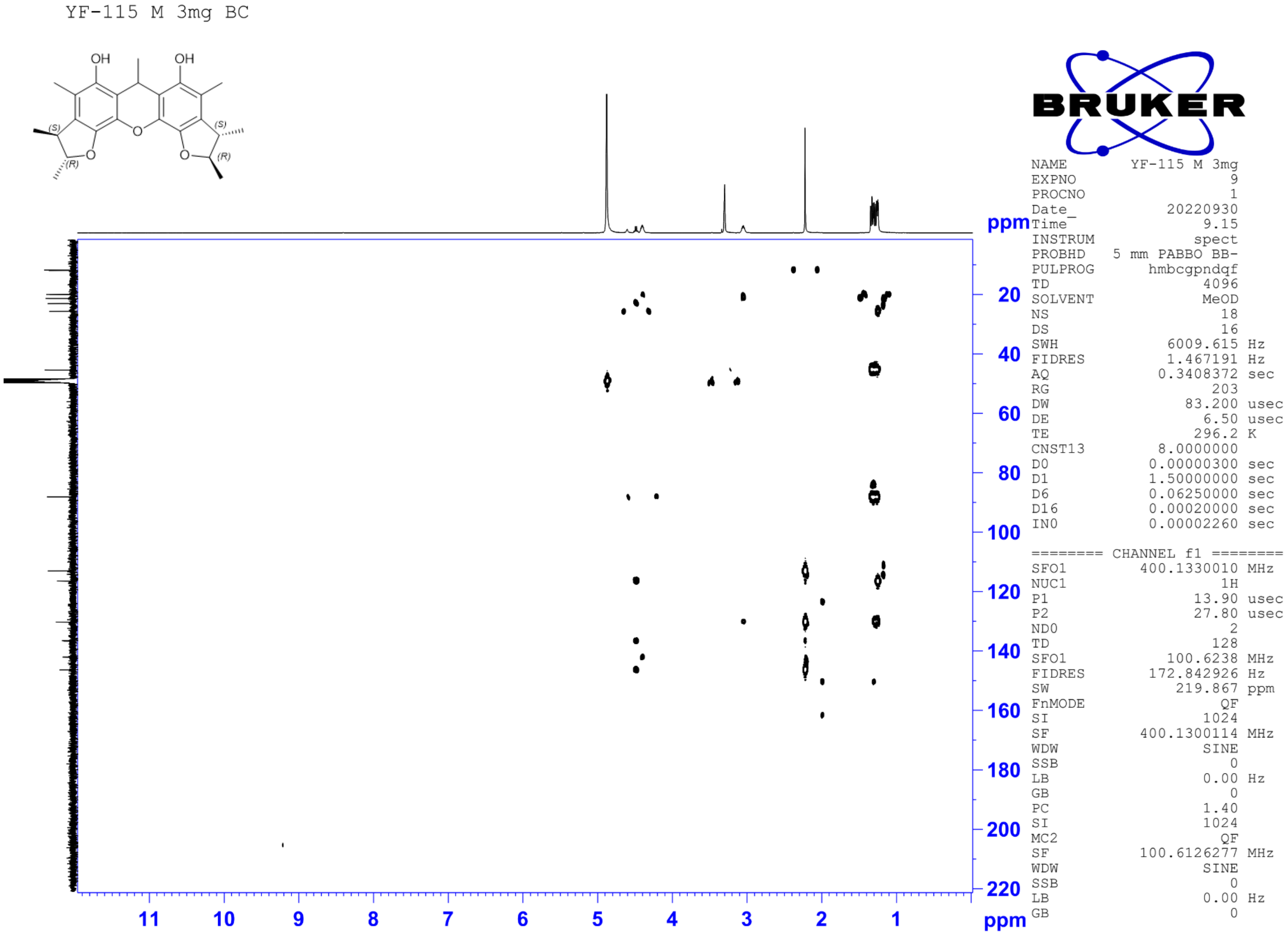

Figure S27. NOESY spectrum of (400 MHz, CD_3OD) compound **4**.

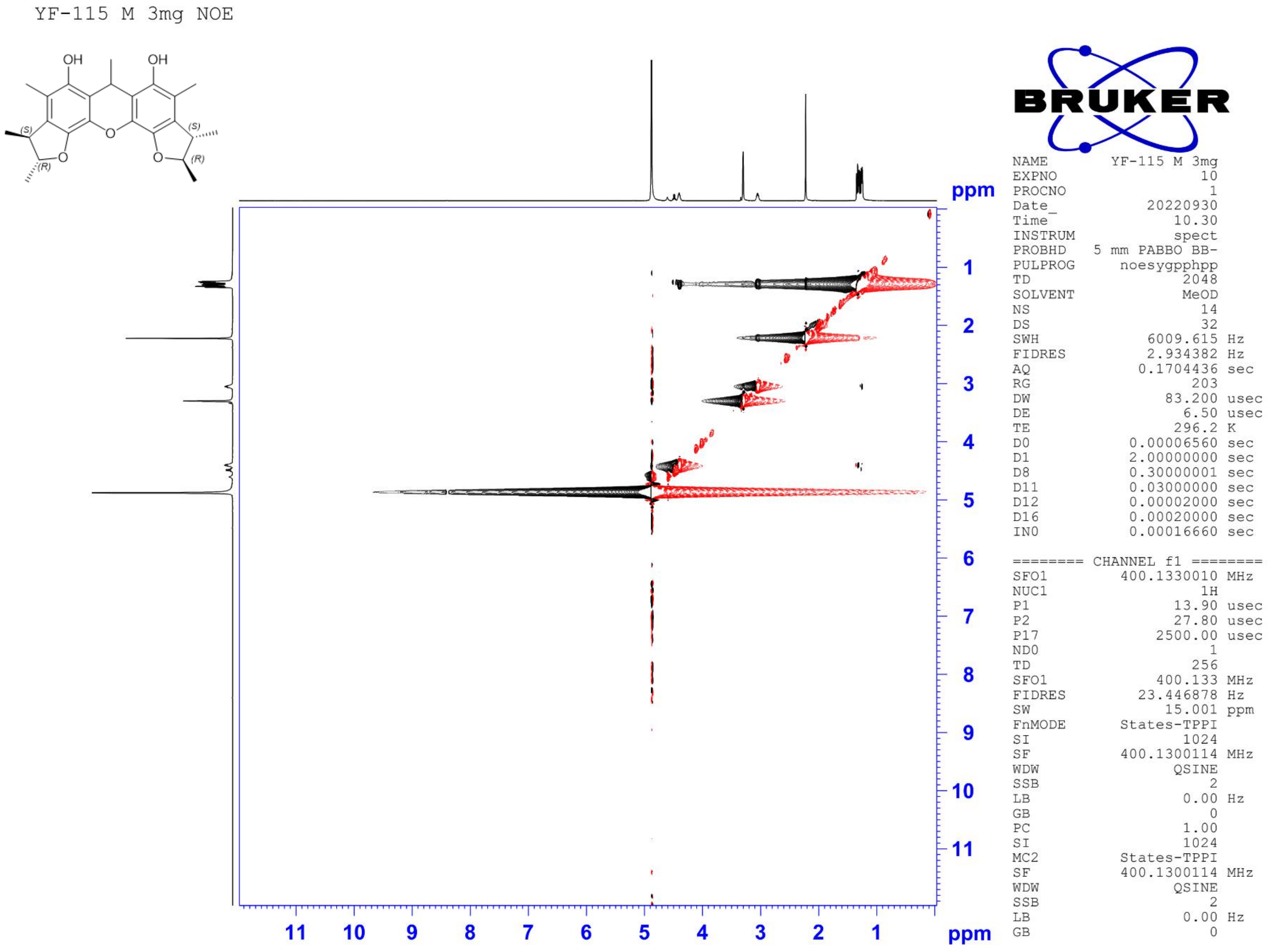

Figure S28. HR-ESI-MS of compound **4**.

Elemental Composition Report **Page 1**

Tolerance = 5.0 mDa / DBE: min = -1.5, max = 50.0
Element prediction: Off
Number of isotope peaks used for i-FIT = 3

Monoisotopic Mass, Even Electron Ions
7288 formula(e) evaluated with 5 results within limits (up to 50 best isotopic matches for each mass)
Elements Used:
C: 0-30 H: 0-30 N: 0-3 O: 0-10 S: 0-6 Cl: 0-8 Br: 0-8

YF-115-N 70 (0.290) Cm (62:96)
1: TOF MS ES-

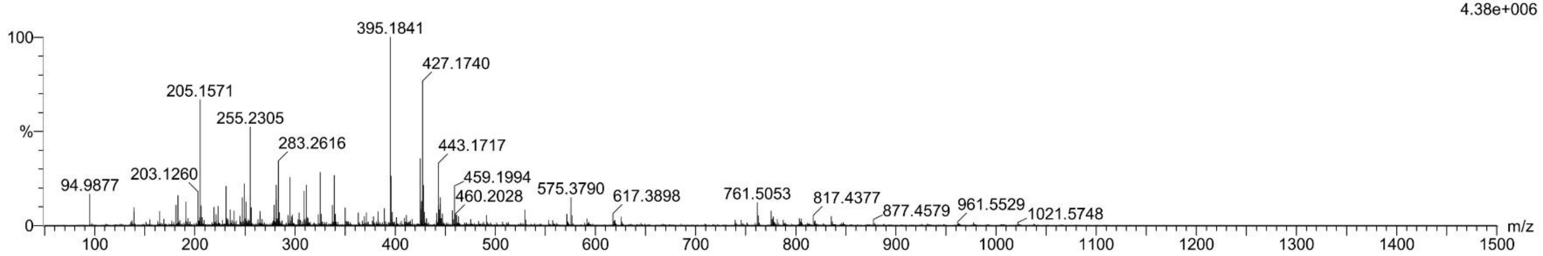

Minimum:	80.00				-1.5				
Maximum:	100.00		5.0	10.0	50.0				

Mass	RA	Calc. Mass	mDa	PPM	DBE	i-FIT	Norm	Conf(%)	Formula
395.1841	100.00	395.1858	-1.7	-4.3	11.5	1915.8	0.124	88.34	C24 H27 O5
		395.1818	2.3	5.8	7.5	1917.9	2.166	11.46	C19 H27 N2 O7
		395.1793	4.8	12.1	11.5	1922.0	6.270	0.19	C23 H27 N2 O2 S
		395.1833	0.8	2.0	15.5	1925.2	9.504	0.01	C28 H27 S
		395.1890	-4.9	-12.4	11.5	1932.1	16.395	0.00	C24 H28 N2 O Cl

Figure S29. ^{1}H NMR spectrum (400 MHz, CD_3OD) of compound **5**.

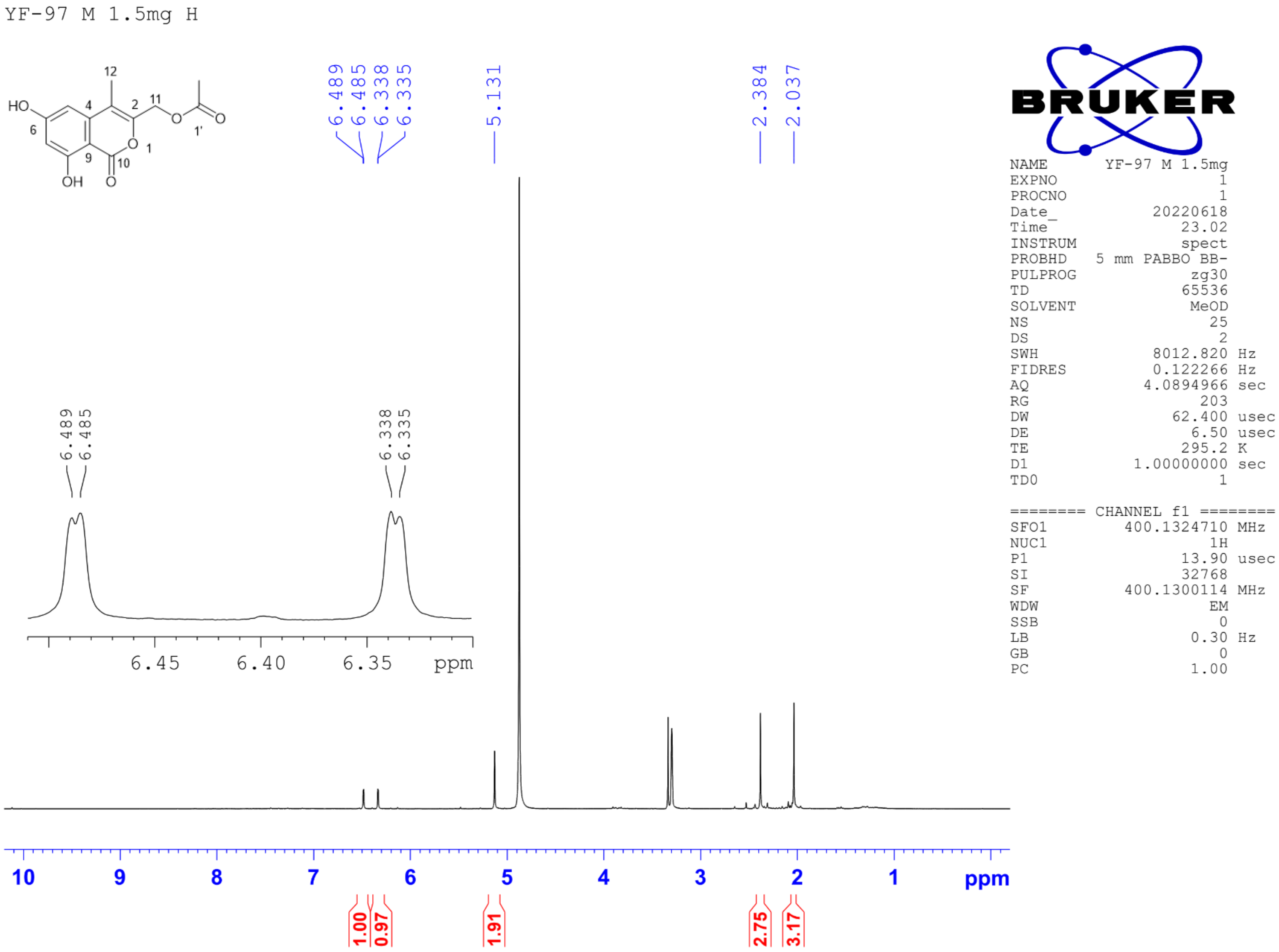

Figure S30. ^{13}C NMR and DEPT spectra (100 MHz, CD_3OD) of compound **5**.

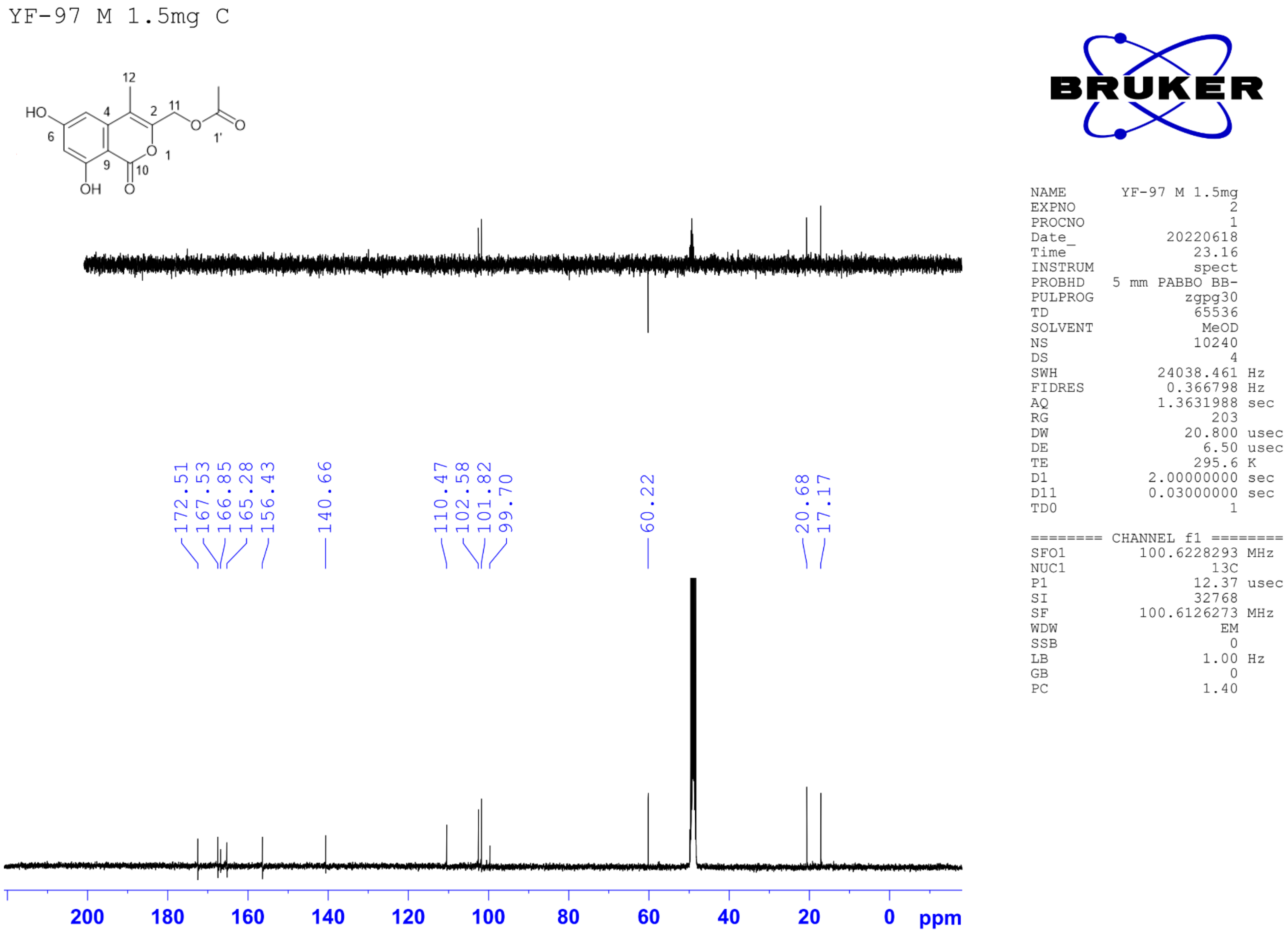

Figure S31. HMQC spectrum (400 MHz, CD_3OD) of compound **5.**

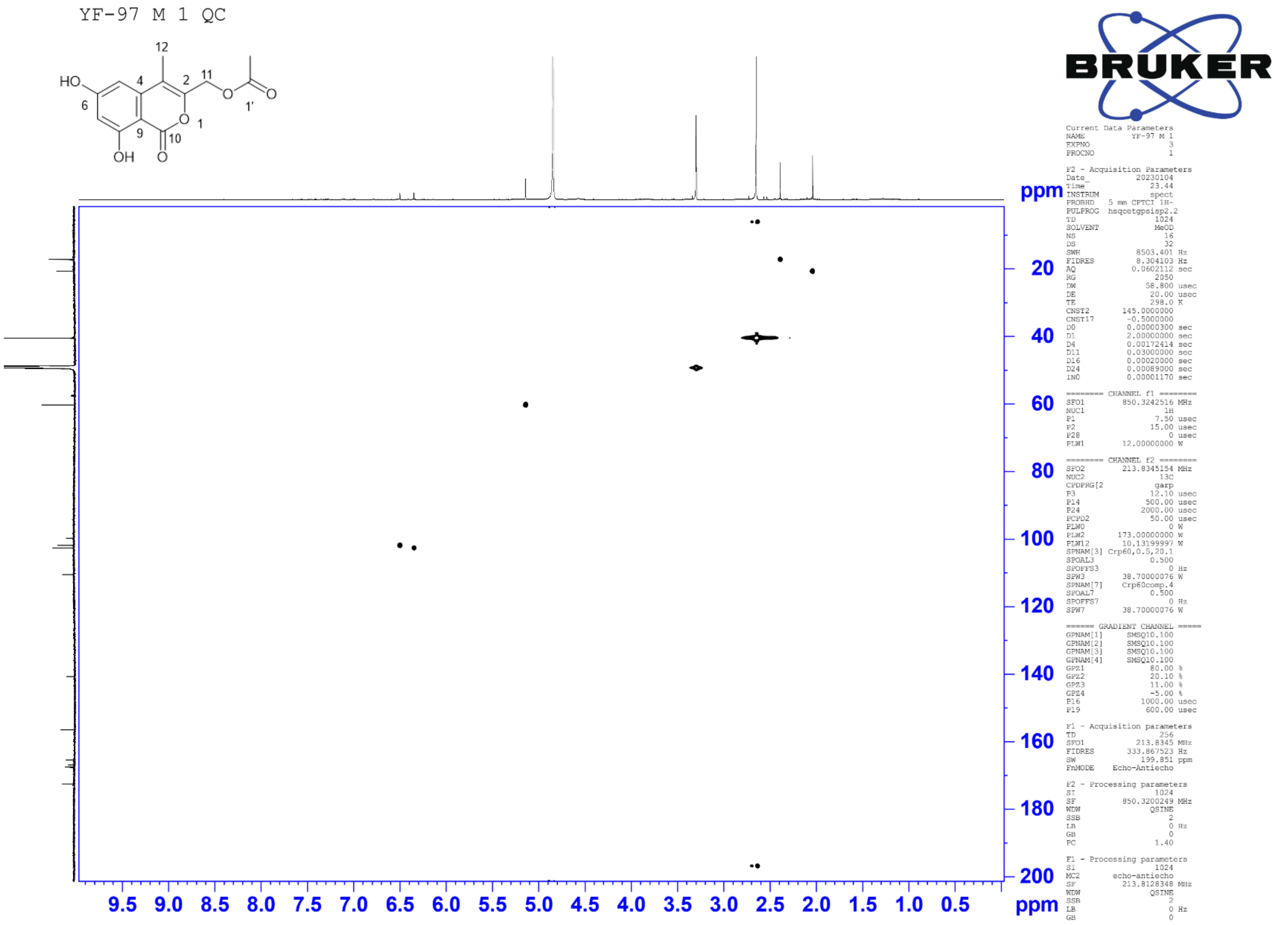

Figure S32. ^{1}H-^{1}H COSY spectrum (400 MHz, CD_3OD) of compound **5**.

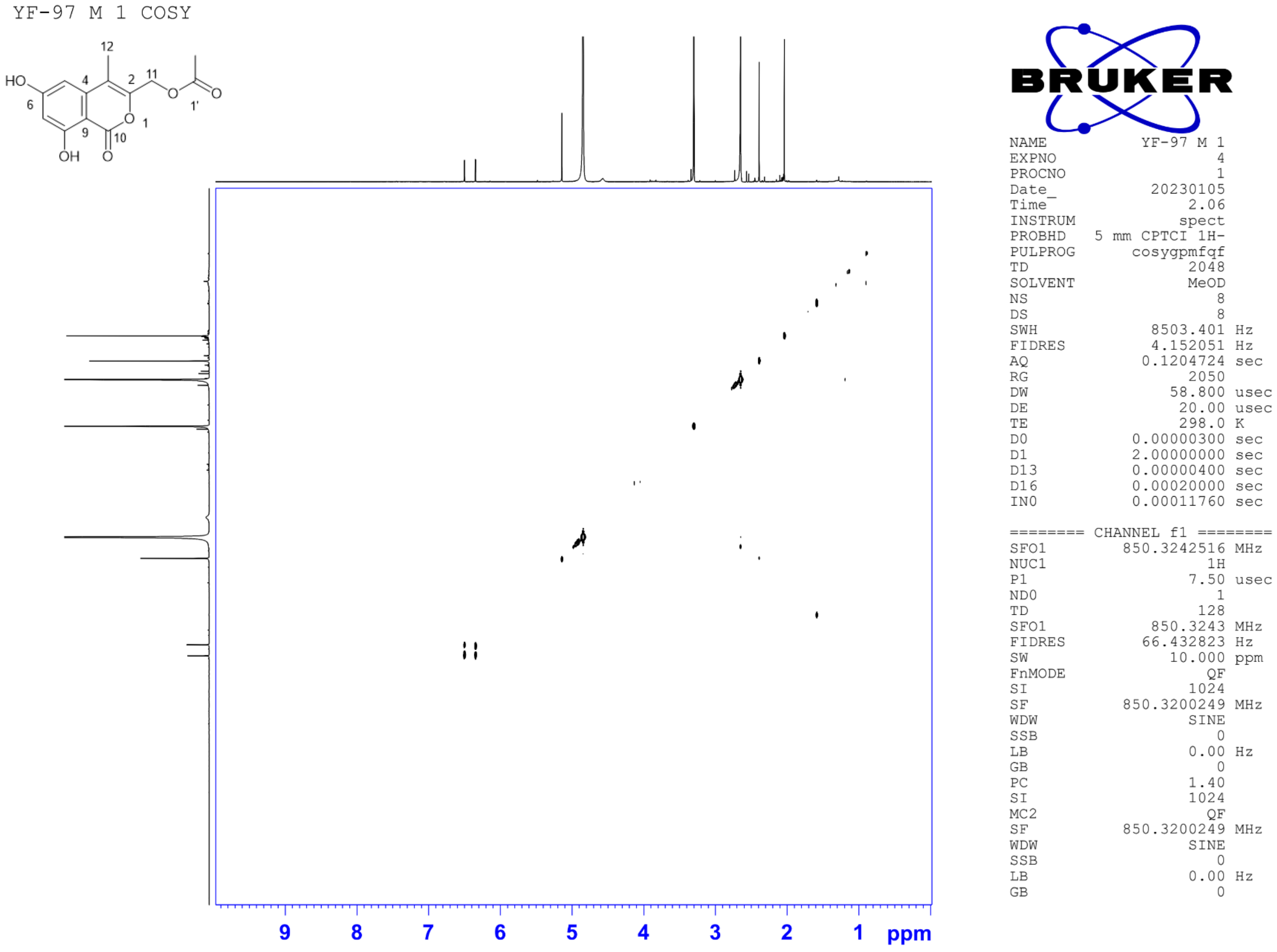

Figure S33. HMBC spectrum (400 MHz, CD_3OD) of compound **5**.

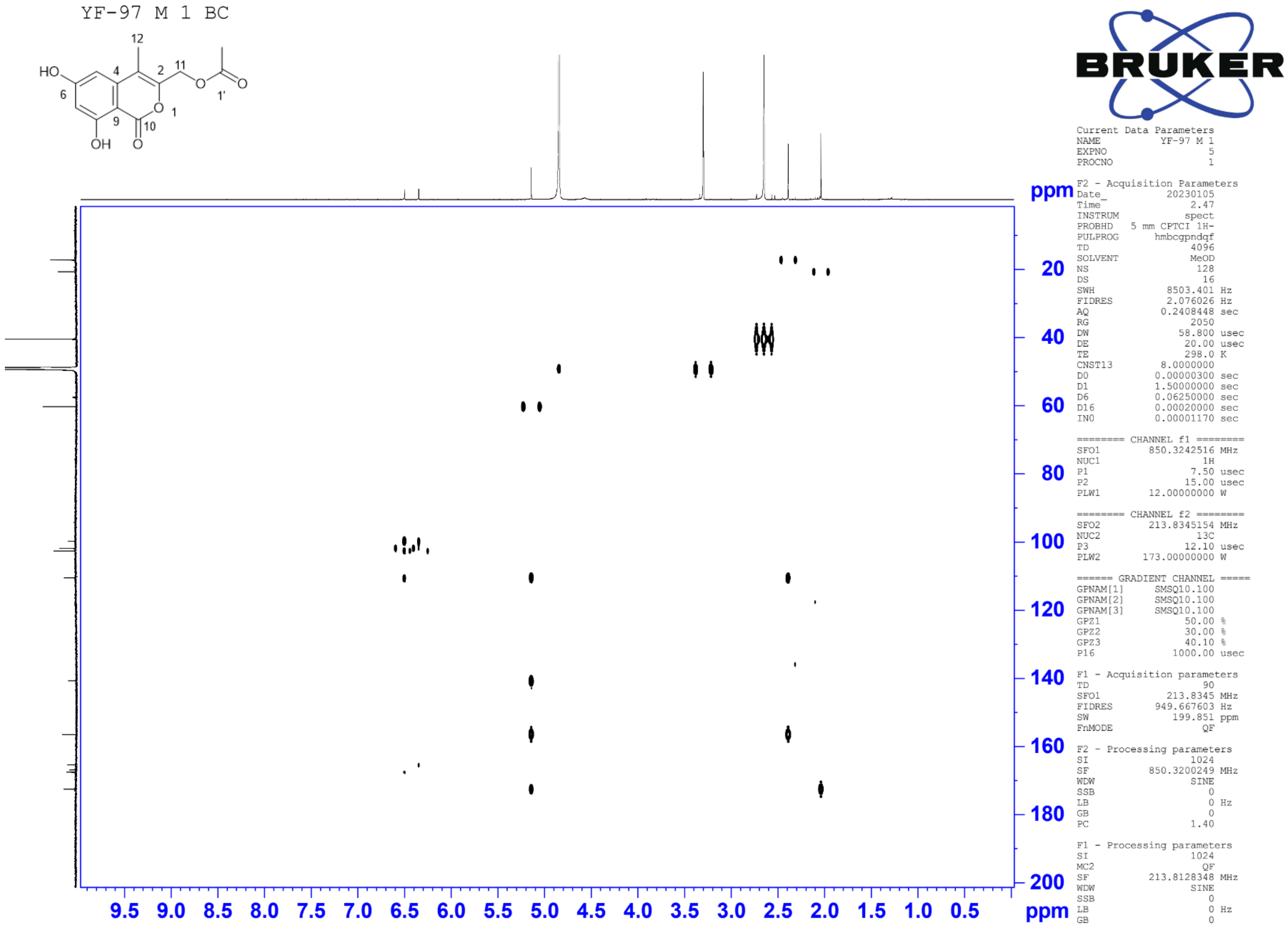

Figure S34. HR-ESI-MS of compound **5**.

Elemental Composition Report

Single Mass Analysis

Tolerance = 30.0 PPM / DBE: min = -1.5, max = 50.0
Element prediction: Off
Number of isotope peaks used for i-FIT = 3

Monoisotopic Mass, Even Electron Ions
99 formula(e) evaluated with 3 results within limits (up to 50 closest results for each mass)
Elements Used:
C: 0-15 H: 0-25 N: 0-5 O: 0-8
YF-97-N 75 (0.308) Cm (70:85)
1: TOF MS ES-

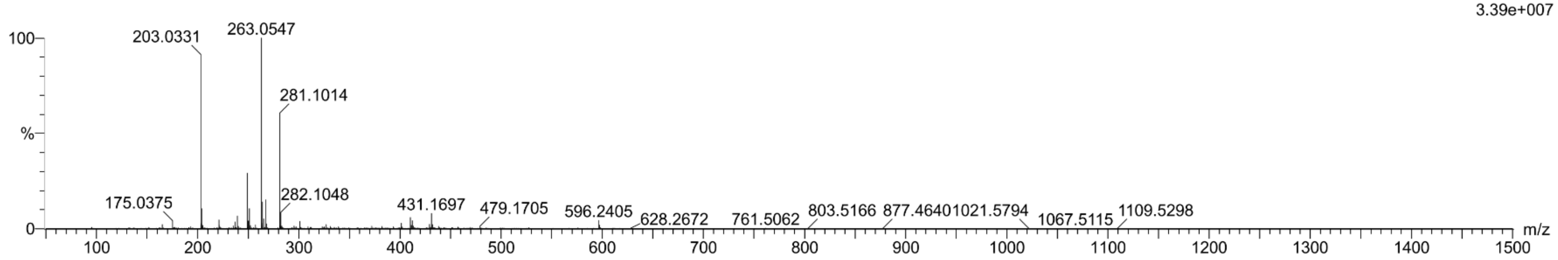

Minimum:				-1.5				
Maximum:		5.0	30.0	50.0				
Mass	Calc. Mass	mDa	PPM	DBE	i-FIT	Norm	Conf(%)	Formula
263.0547	263.0556	-0.9	-3.4	8.5	1939.8	0.491	61.20	C13 H11 O6
	263.0569	-2.2	-8.4	13.5	1940.7	1.399	24.68	C14 H7 N4 O2
	263.0515	3.2	12.2	4.5	1941.2	1.957	14.13	C8 H11 N2 O8

Figure S35. ^{1}H NMR spectrum (400 MHz, CD_2Cl) of compound **6**.

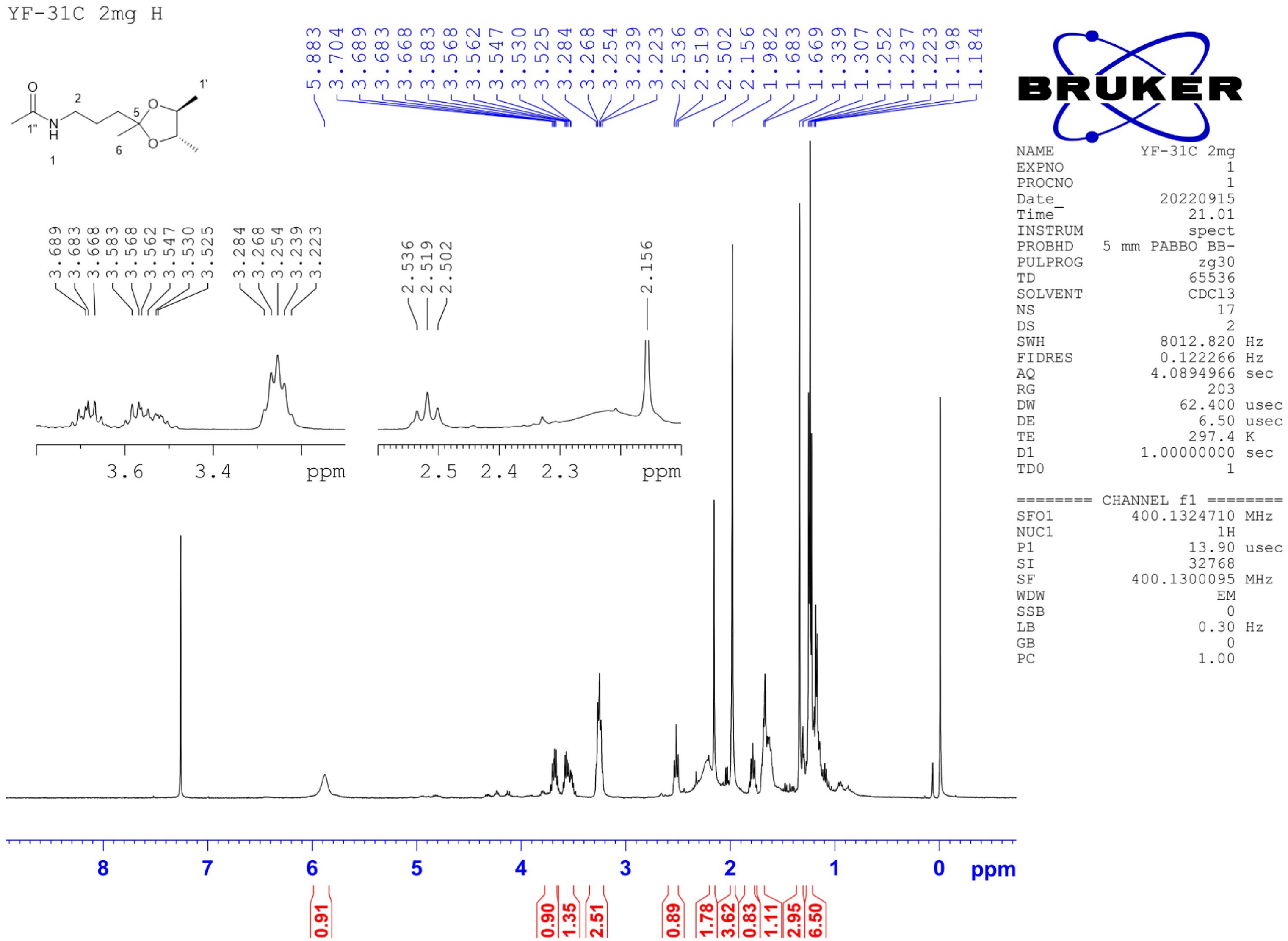

Figure S36. ^{13}C NMR and DEPT spectra (100 MHz, CD_2Cl) of compound **6**.

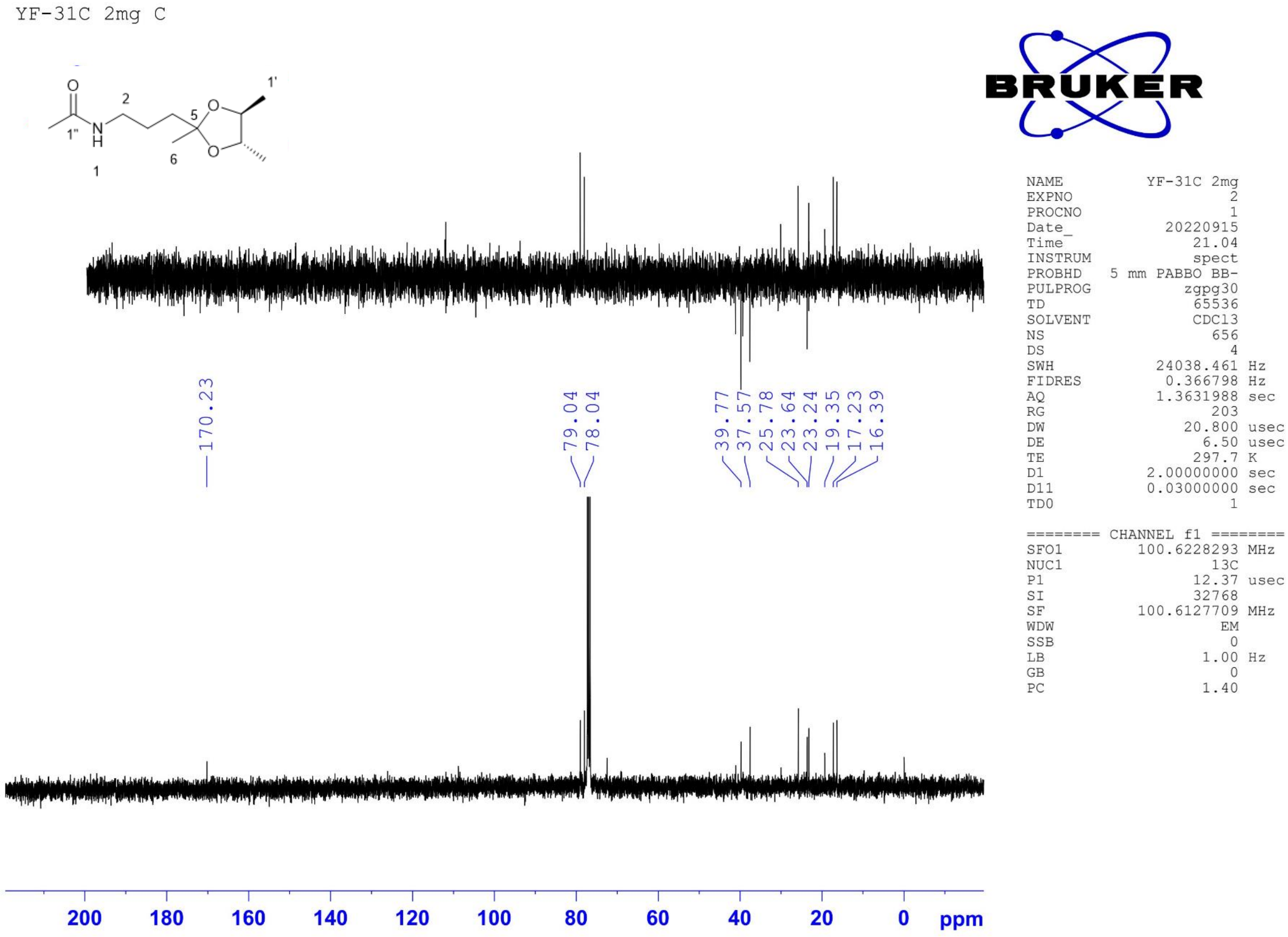

Figure S37. HMQC spectrum (400 MHz, CD_3OD) of compound **6**.

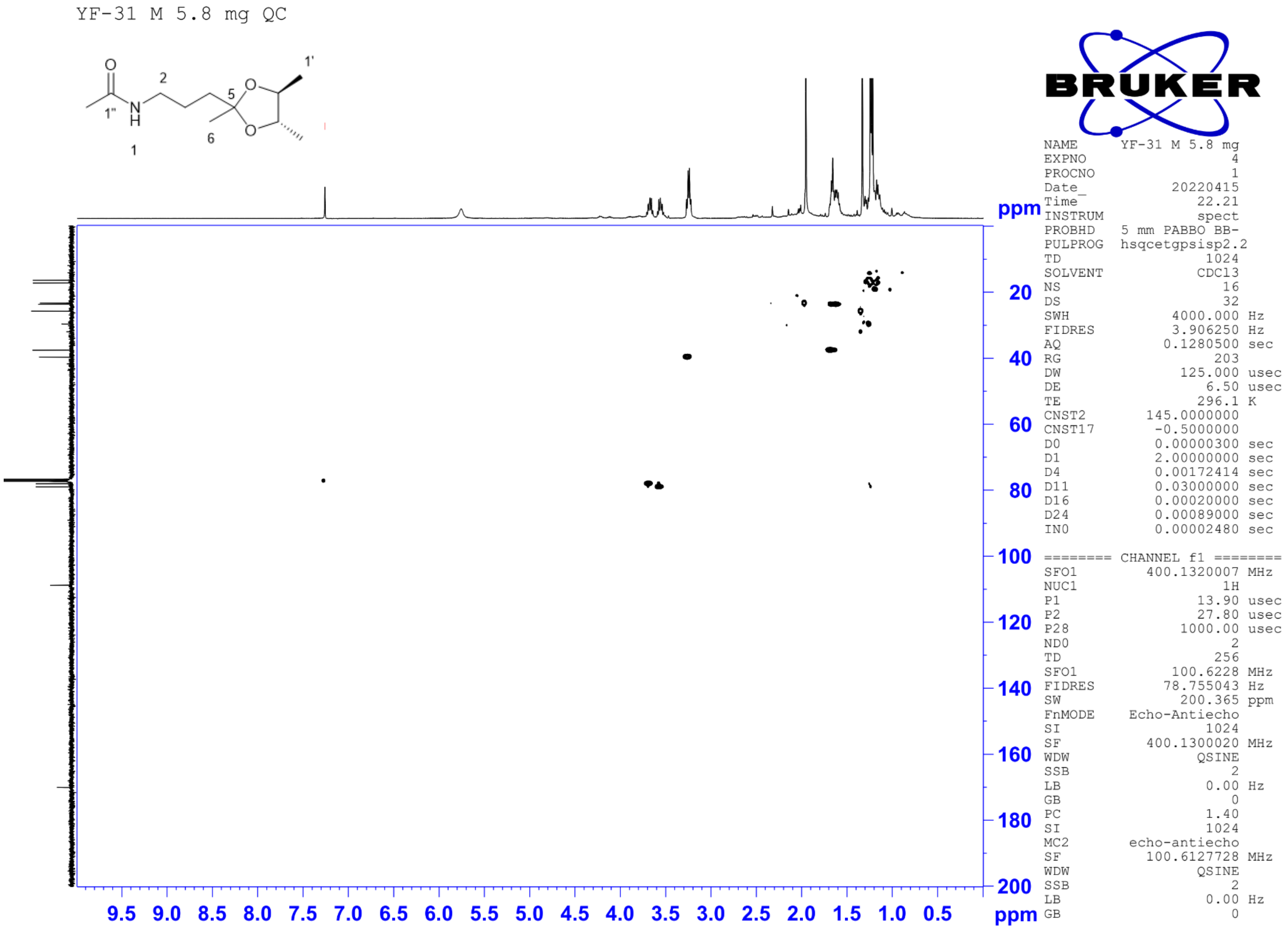

Figure S38. ^{1}H-^{1}H COSY spectrum (400 MHz, CD_3OD) of compound **6**.

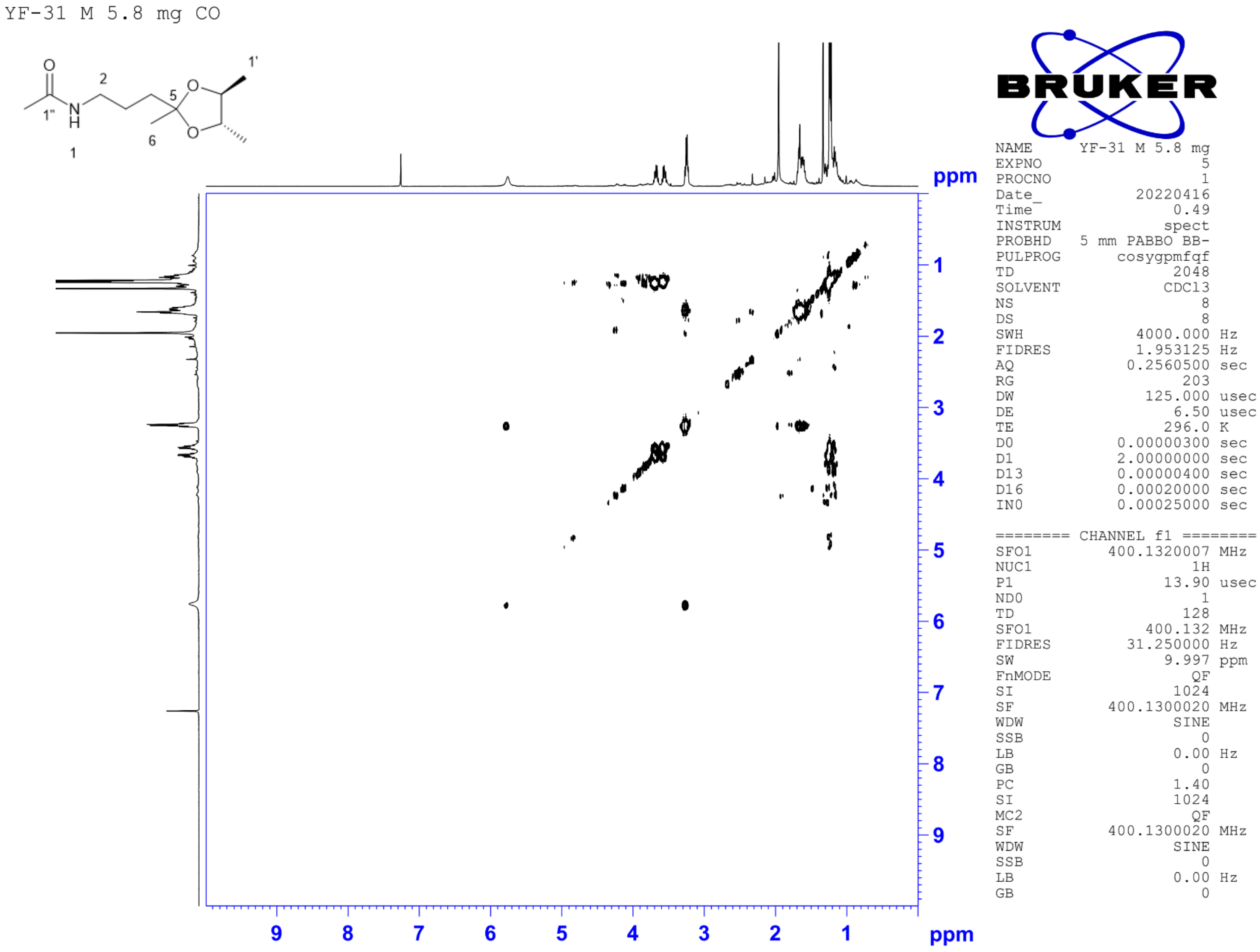

Figure S39. HMBC spectrum (400 MHz, CD_3OD) of compound **6**.

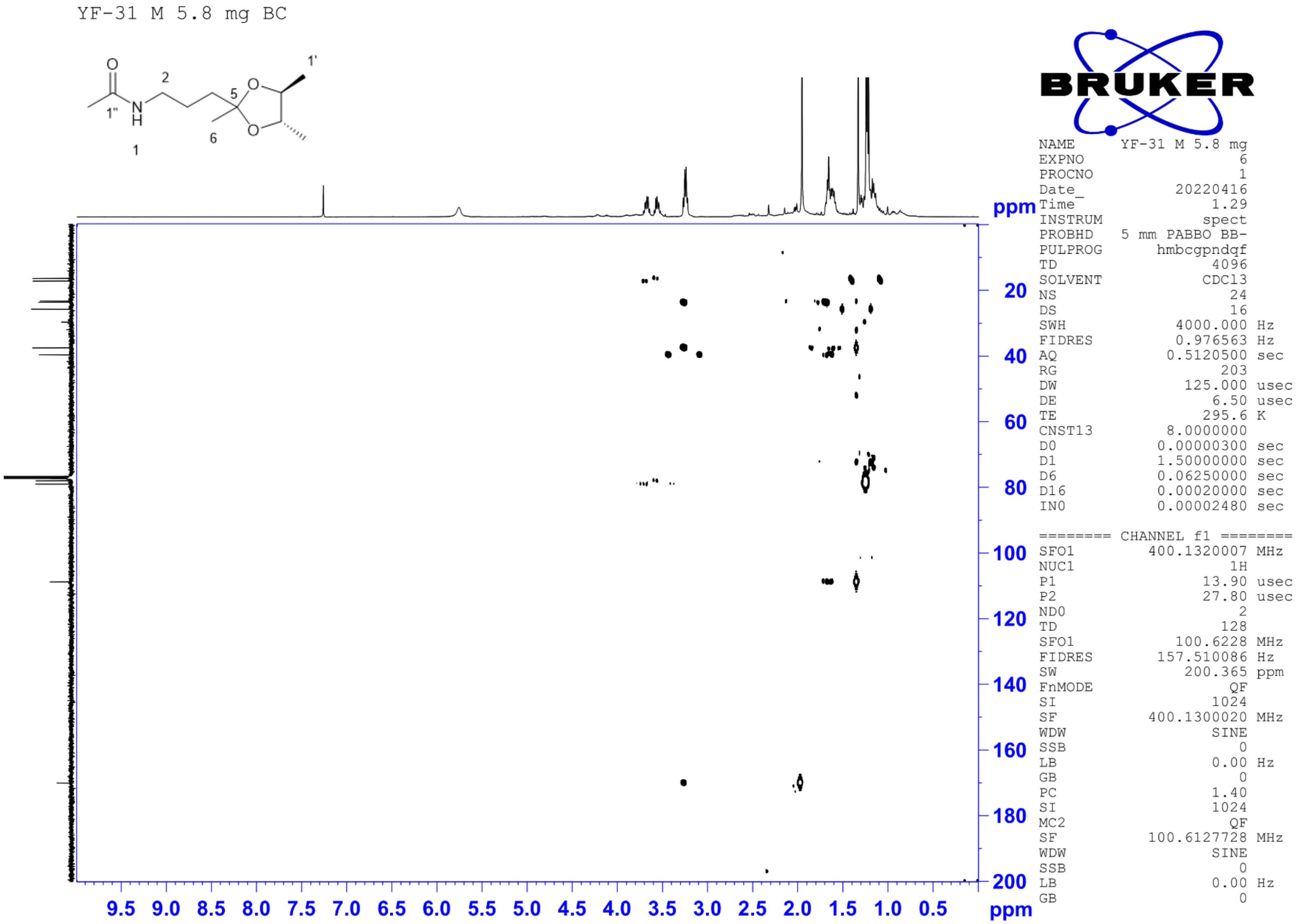

Figure S40. NOESY spectrum of (400 MHz, CD_3OD) compound **6.**

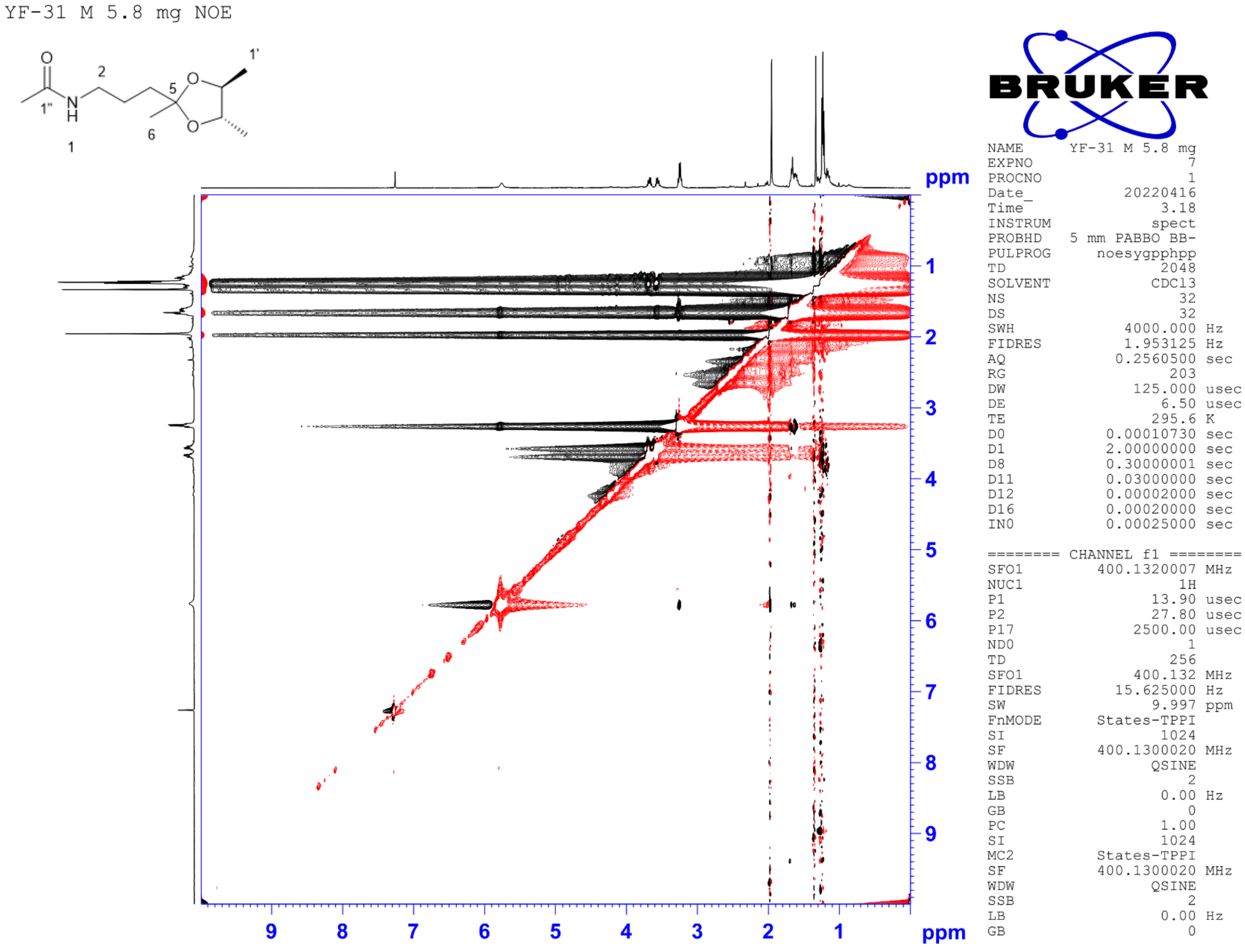

Figure S41. HR-ESI-MS of compound **6.**

Elemental Composition Report

Single Mass Analysis

Tolerance = 30.0 PPM / DBE: min = -1.5, max = 50.0
Element prediction: Off
Number of isotope peaks used for i-FIT = 3

Monoisotopic Mass, Even Electron Ions
144 formula(e) evaluated with 3 results within limits (up to 50 closest results for each mass)
Elements Used:
C: 0-15 H: 0-25 N: 0-5 O: 0-5 Na: 0-1

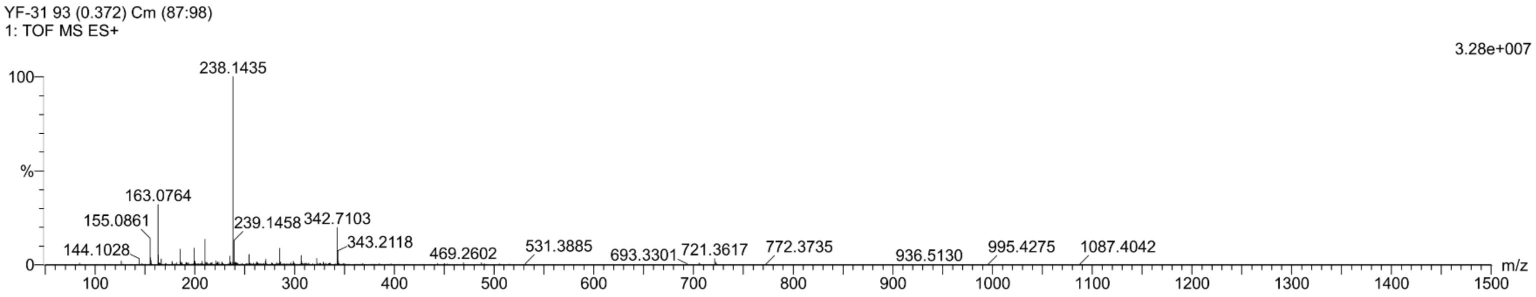

Minimum:				-1.5				
Maximum:		5.0	30.0	50.0				
Mass	Calc. Mass	mDa	PPM	DBE	i-FIT	Norm	Conf(%)	Formula
238.1435	238.1443	-0.8	-3.4	4.5	2068.0	6.456	0.16	C13 H20 N O3
	238.1419	1.6	6.7	1.5	2061.5	0.008	99.22	C11 H21 N O3 Na
	238.1403	3.2	13.4	0.5	2066.6	5.083	0.62	C8 H20 N3 O5

Figure S42. Calculated and experimental ECD spectra of 2.

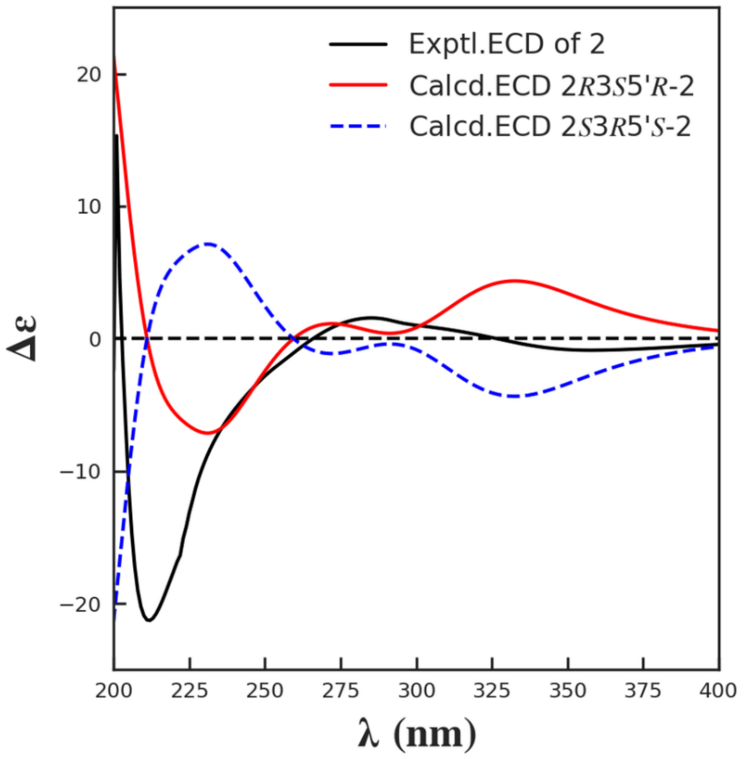

Supplement: Supplementary file 1 [file marinedrugs-21-00504-s001.zip › marinedrugs-2614435-supplementary.pdf]
